# Antioxidant activities, dietary nutrients, and yield potential of bitter gourd (*Momordica charantia* L.) lines in diverse growing environments

**DOI:** 10.3389/fnut.2024.1393476

**Published:** 2024-08-06

**Authors:** Gograj Singh Jat, Tusar Kanti Behera, Awani Kumar Singh, Ram Swaroop Bana, Deepak Singh, Samarth Godara, Umesh K. Reddy, P. Gangadhara Rao, H. Ram, N. D. Vinay, Sachin Kumar, Bhoopal Singh Tomar

**Affiliations:** ^1^Division of Vegetable Science, ICAR–Indian Agricultural Research Institute, New Delhi, India; ^2^ICAR–Indian Institute of Vegetable Research, Varanasi, Uttar Pradesh, India; ^3^Centre for Protected Cultivation Technology, ICAR–Indian Agricultural Research Institute, New Delhi, India; ^4^Division of Agronomy, ICAR–Indian Agricultural Research Institute, New Delhi, India; ^5^ICAR–Indian Agricultural Statistics Research Institute, New Delhi, India; ^6^Department of Biology, Gus R. Douglass Institute, West Virginia State University, Institute, WV, United States; ^7^Department of Vegetable Science, Dr. YSR Horticultural University, West Godavari, Andhra Pradesh, India; ^8^ICAR–Central Institute for Arid Horticulture, Bikaner, Rajasthan, India; ^9^ICAR–National Institute of Biotic Stress Management, Raipur, Chhattisgarh, India

**Keywords:** antioxidants, micronutrients, protected cultivation, GYT biplot, bitter gourd

## Abstract

The biotic and abiotic stresses cause a significant decline in the yield and fruit quality traits, including antioxidants and minerals, of bitter gourd when grown in open fields. Protected cultivation technology has emerged to minimize such stresses. We investigated the effect of diverse environments (hi-tech greenhouse, naturally ventilated polyhouse, insect-proof net-house, and open field) and breeding lines on earliness, yield potential, antioxidant activities, and dietary nutrients. In the GYT analysis, 12 treatment combinations involving four growing environments and three breeding lines of bitter gourd were examined. The 3-year study suggested that the cultivation of bitter gourd crops in an insect-proof net house (NH) showed superior performance in earliness, yield-attributing traits, antioxidant activities, and dietary nutrients, followed by a naturally ventilated polyhouse (NP). However, NH was on par with NP and significantly better than the open-field-grown crop. The GYT biplot analysis highlighted that the combinations of NH and Pusa Rasdar outperformed and were the most stable treatments for all the traits investigated, followed by NH in conjunction with S32 and S57 lines. This study suggests that growing bitter gourd in protected environments is the optimal strategy to achieve early market prices and improve the yield and nutritional quality of the fruits.

## 1 Introduction

Bitter gourd (*Momordica charantia* L.) is an industrially important, phytochemical-rich, health-promoting vegetable crop of the Cucurbitaceae family (1). Over 60 phytonutrients have been reported in different parts of this pharmaceutically significant crop, known for its potential therapeutic properties in treating over 30 deadly diseases, including diabetes and cancer (1–3). Its fruits are an excellent source of carotenoids, including β-carotene, zeaxanthin, and lycopene (at the ripe stage), and lutein and α-carotene (principally at the immature stage) (4, 5). Other antioxidants in bitter gourd plants include vitamin C, vitamin E, phenolic acids, and organosulfur compounds (5–7). The cultivated area of bitter gourd is increasing every year across the globe, including in European countries and the United States of America (8).

Nevertheless, the yield and nutritional quality of bitter gourd significantly diminish due to a range of biotic stressors such as fruit fly, whitefly, aphids, and complex viral diseases, as well as abiotic stresses such as temperature fluctuations during the flowering stage leading to sex modification. Such declines are especially prominent during the rainy and post-rainy seasons (9, 10). These challenges in open-field-grown bitter gourd crops have necessitated the development of climate-resilient and sustainable alternative methods for growing the crop (11).

Furthermore, to overcome biotic stresses, the application of multiple agrochemicals in open-field cultivation becomes necessary. This practice not only increases production costs but also increases the likelihood of residual toxicity, especially in freshly produced fruits. This is particularly risky when diabetics use freshly harvested fruits for juice extraction (5, 11). Hence, switching to a production alternative such as protected cultivation becomes imperative to tackle these concerns (9). The significance of protected cultivation for vegetables lies in the production of safer and healthier yields, primarily attributed to reduced reliance on plant-protection chemicals, principally due to minimal pest infestations (12).

Moreover, protected cultivation offers opportunities to grow vegetables during the off-season with better quality produce and yields that are 2–3 times higher compared to open field conditions (13, 14). However, there is a lack of information on the effects of protected structures on antioxidants and mineral nutrients in bitter gourd fruits, specifically in sub-tropical semiarid agroecologies.

Another notable bottleneck in bitter gourd cultivation is the monoecious nature of current bitter gourd varieties, characterized by a high male-to-female flower ratio of 15:1 and late flowering traits, resulting in the wastage of vast land resources and very low yield potential (8, 15, 16). To overcome this obstacle, the Indian Agricultural Research Institute, New Delhi, has developed three predominantly gynoecious advanced lines with a female-to-male flower ratio (2:1), coupled with earliness and greater yields. However, systematic information on the growing conditions of these new lines and the suitable varieties/hybrids for different protected structures is not available (17). Therefore, the current investigation was conducted to test the effectiveness of varied growing scenarios on different bitter gourd breeding lines with respect to earliness and yield traits, and to quantify the antioxidant and dietary nutrients of these breeding lines in diverse growing environments.

## 2 Materials and methods

### 2.1 Experimental site

A 3-year (2018–2020) experiment was conducted at the Center for Protected Cultivation Technology and Division of Vegetable Science, ICAR-Indian Agricultural Research Institute, New Delhi (28° 4' N, 77° 12' E, 228.6 m altitude) on sandy loam Inceptisol soils. Using a core sampler, composite soil samples were taken at a depth of 0–150 mm before transplanting seedlings to analyze the soil's physical and chemical properties. The soil of the experimental field was slightly alkaline with low organic carbon and plant-available nitrogen, whereas plant-available phosphorus and potassium were moderate. Within the composite soil samples, the plant-extractable zinc, iron, manganese, and copper were 0.56, 4.34, 5.31, and 1.62 mg kg ^−1^, respectively. The climate of the study area was semiarid, with high diurnal and seasonal temperature variations. The weather data recorded in open fields and protected structures during the cropping period (2018, 2019, and 2020) in the standard meteorological week (SMW) are presented in Supplementary Table 1. Temperature and relative humidity were recorded with an Assmann psychrometer (Model MR-58, Hisamatsu, Tokyo, Japan), and photosynthetically active radiation (PAR) was recorded with a line quantum sensor (MQ-301, Series#1178, Apogee, Logan, UT, USA).

### 2.2 Experimental materials

The experiment was conducted in four diverse growing environments, including three protected structures (a hi-tech greenhouse, a naturally ventilated polyhouse, and an insect-proof net house) and an open field. All the protected structures were oriented in an east-west direction. In the hi-tech greenhouse, all climatic parameters were fully controlled to meet the crop requirements. The naturally ventilated polyhouse structure was framed with an insect-proof net (a ventilated area covered with a 40-mesh net) and covered with transparent plastic (a 200-micron UV-stabilized 5-layer high-density polyethylene sheet with 90% light transmission) to trap sunlight during the winter season. The insect-proof net house was covered with a UV-stabilized insect-proof net of 40 mesh for the effective control of pests and diseases. Drip-fertigation facilities were laid in all the structures.

**Figure 1:**
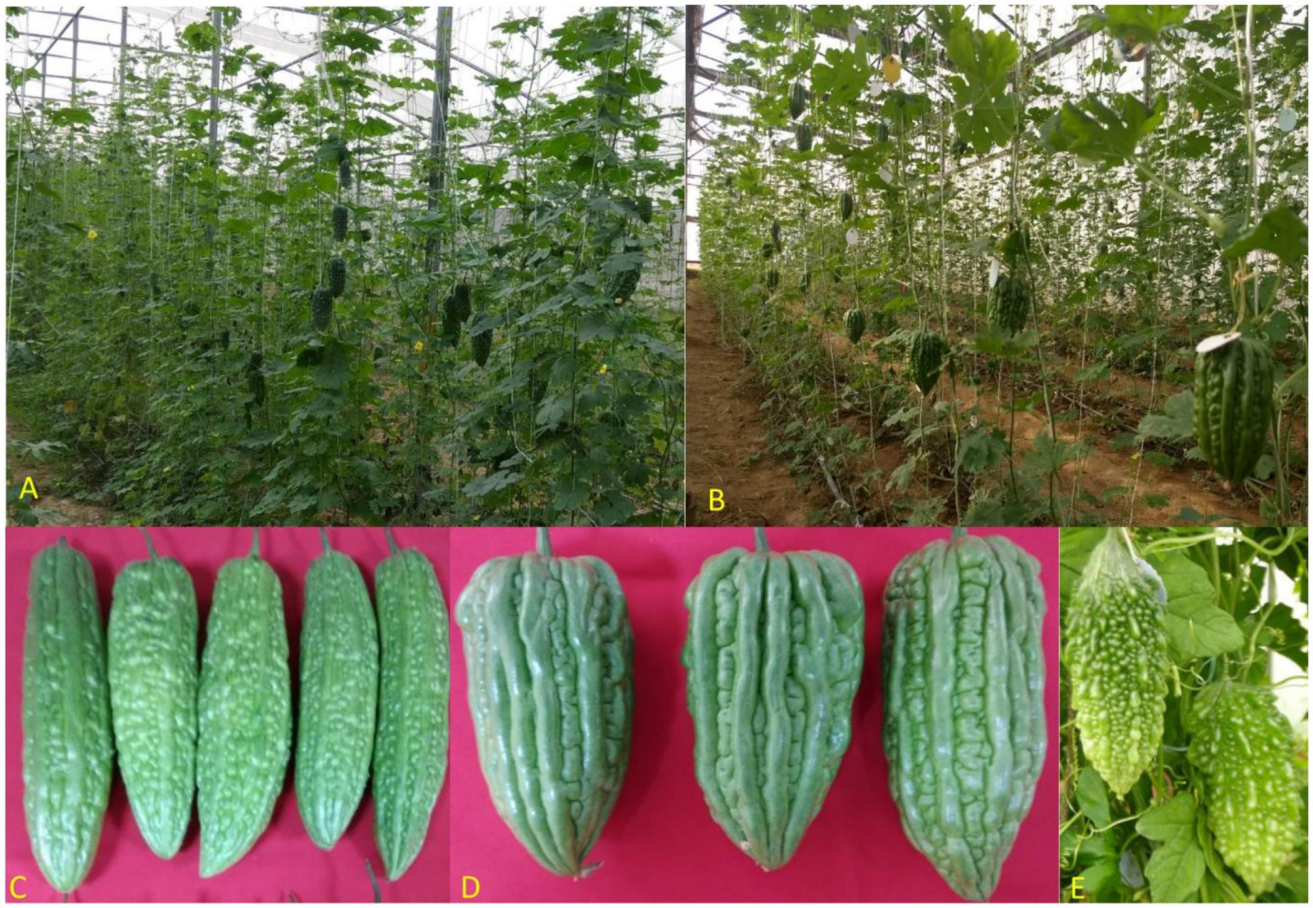
An overview of the 3-year bitter gourd experiment conducted in different environments. **(A)** Insect-proof net house (use of a 40-mesh insect-proof net and no environmental control); **(B)** High-tech greenhouse (temperature and relative humidity are maintained); **(C)** Fruits of S32 (attractive green, uniform, glossy, cylindrical, and straight); **(D)** Pusa Rasdar (continuous ridges, smooth surface, juicy, and capsicum shaped fruits); and **(E)** S57 (discontinuous ridges and blunt tubercles on fruit surface).

The experimental genotypes consisted of one released variety (Pusa Rasdar) and two advanced breeding lines, S32 and S57 (Figure 1). These three varieties/lines are highly stable and predominantly gynoecious in nature (higher female: male flower ratio of 2:1), which makes them very suitable for protected cultivation through manual pollination due to the higher number of female flowers per plant and large flower size, requiring only the dusting of pollen on the stigma of the female flower. One male flower is sufficient to pollinate 3 to 4 female buds.

Pusa Rasdar is an exceptionally early variety characterized by juicy, smooth, non-prickled, tender-skinned fruits that are fleshy and exhibit an attractive dark green color. S32 has a higher female-to-male flower ratio, with fruits that are attractive green, uniform, glossy, cylindrical, and straight. S57 is another predominantly gynoecious line with attractive green-colored fruits with broken ridges on the fruit surface (Figure 1).

### 2.3 Treatment details

The experiment was conducted in a split-plot design with three replications, covering a gross area of 1,000 m^2^ in each environment. A uniform dose of NPK fertilizers (80 kg N ha^−1^, 50 kg P_2_O_5_ ha^−1^, and 40 kg K_2_O ha^−1^) was applied across all growing environments. In the protected structures, water-soluble fertilizers were applied using a drip irrigation system, whereas, in the open field, fertilizers were broadcast in the hill and channel system for the bitter gourd crop. The Genotype × Yield × Traits (GYT) biplots of different characters are given in Table 1, and the details of treatment combinations (growing environments and breeding lines/variety) are presented in Table 2.

**Table 1:** Different yields by traits used for GYT biplots of different characters.

**GYT**	**Trait**	**Trait short form**	**Yield by trait (GYT trait)**	**Formula used for GYT**
Earliness character	Node number of first female flower	NNFFF	Yield × NNFFF	Y/NNFFF
	Days until the first female flower anthesis	DFFFA	Yield × DFFFA	Y/DFFFA
	Days until the first fruit harvest	DFFH	Yield × DFFH	Y/DFFH
Yield contributing character	Fruit length (cm)	FL	Yield × FL	Y × FL
	Fruit diameter (cm)	FD	Yield × FD	Y × FD
	Average fruit weight (g)	FW	Yield × FW	Y × FW
	Number of fruit/plants	F/P	Yield × F/P	Y × F/P
Antioxidants character	Juice content (ml/500 g)	JC	Yield × JC	Y × JC
	DPPH (mg/100 g)	DPPH	Yield × DPPH	Y × DPPH
	Chlorophyll (mg/100 g)	Chl	Yield × Chl	Y × Chl
	Vitamin-C (mg/100 g)	Vit_C	Yield × Vit_C	Y × Vit-C
	Carotenoid (mg/100 g)	Carot	Yield × Carot	Y × Carot
	Saponin (μg/g)	Sap	Yield × Sap	Y × Sap
	Charantine (μg/g)	Charan	Yield × Charan	Y × Charan
Minerals character	Mn (mg/100 g)	P	Yield × P	Y × P
	Zn (mg/100 g)	K	Yield × K	Y × K
	Fe (mg/100 g)	Mn	Yield × Mn	Y × Mn
	P (mg/100 g)	Zn	Yield × Zn	Y × Zn
	K (mg/100 g)	Fe	Yield × Fe	Y × Fe

Yield is denoted by Y.

**Table 2:** Details of treatments applied to the bitter gourd crops.

**Treatment**	**Treatment combination**	**Treatment details**
**Environment-I (high-tech green house with environmental**		
**control)**		
T1	HG_PR	Hi-tech Greenhouse and Pusa Rasdar
T2	HG_S32	Hi-tech Greenhouse and S32
T3	HG_S57	Hi-tech Green House and S57
**Environment-II (naturally ventilated polyhouses with partial**		
**environmental control)**		
T4	NP_PR	Naturally ventilated Polyhose and Pusa Rasdar
T5	NP_S32	Naturally ventilated Polyhose and S32
T6	NP_S57	Naturally ventilated Polyhose and S57
**Environment-III (insect-proof net houses with natural**		
**environment)**		
T7	NH_PR	Net house and Pusa Rasdar
T8	NH_S32	Net House and S32
T9	NH_S57	Net House and S57
**Environment-IV (open fields: no environmental control)**		
T10	OF_PR	Open Field and Pusa Rasdar
T11	OF_S32	Open Field and S32
T12	OF_S57	Open Field and S57

### 2.4 Nursery raising and transplanting

The seedlings were raised in 1.5-inch conical plug trays using a soil-less media mixture of cocopeat, vermiculite, and perlite in a 3:1:1 ratio at a high-tech nursery facility. After sowing the seeds, trays were placed in a seed germination chamber at 20°C and 100% relative humidity until germination started. Subsequently, they were moved to a high-tech vegetable nursery greenhouse. The 25-day-old seedlings were transplanted onto raised beds in all three protected environments and into a hill and channel system at a distance of 45 cm apart on the slope of channels prepared at a distance of 2.5 m in open field conditions. Transplanting was conducted on both sides of the hills. Among the 20 plants, 10 were randomly selected in each replication for observations on earliness, yield, antioxidants, and mineral nutrients.

### 2.5 Crop management and the cost of hand pollination

In the experimental area, 50% nitrogen fertilizer and 100% phosphorus fertilizer, and potassium fertilizer were applied before transplanting the seedlings. The remaining nitrogen fertilizer was applied in two equal, split doses at 30 and 60 days after transplanting. To control weeds, pendimethalin herbicide was applied at 0.75 kg active ingredients ha^−1^ as a pre-emergence application, followed by two manual weedings just before the application of the second and third doses of nitrogen fertilizer.

To protect the crop from fruit flies, aphids, and white flies in the open field, the recommended insecticides were sprayed using a battery-powered knapsack sprayer at regular intervals as needed. In protected structures, the spray of insecticides was avoided due to a negligible infestation. Hand pollination was carried out during the morning hours (7:00 am to 9:00 am) in protected structures, while natural pollination was allowed in open-field conditions throughout the experiments. Bitter gourd fruits were harvested at regular intervals at the edible maturity stage. For antioxidants and mineral nutrients analysis, the fruits were harvested at the peak fruiting stage from randomly selected 30 plants within each experimental area.

The cost of hand pollination is an important aspect of the protected cultivation of bitter gourd. The flowering period of bitter gourd fruits lasts only 40–45 days. For this period, we hired only one skilled laborer for one and a half months to pollinate a 1,000 m^2^ area in each protected structure, which generally took 3 h (07:00 am−10:00 am). The cost of hand pollination for a month was only Rs. 10,000. The total cost of pollination for the entire flowering period was Rs. 15,000 (~$200). However, this cost can be compensated by the benefits of early market prices, off-season production, better fruit quality, and the absence of pesticide residues. In contrast, open-field-grown bitter gourd crops need frequent pesticide sprays to control pests such as fruit flies, red pumpkin beetles, and several viral diseases.

### 2.6 Earliness and yield parameters

Key earliness traits in bitter gourd, such as the node number of the first female flower (NNFFF), days to the first female flower anthesis (DFFFA), and days to the first fruit harvest (DFFH), were recorded in randomly selected 10 plants in each replication, excluding border rows. Yield and its related traits (fruit length, fruit diameter, average fruit weight, number of fruits per plant, and yield per plant) were recorded at the edible maturity stage.

### 2.7 Estimating antioxidants and mineral nutrient concentrations in bitter gourd fruits

The juice quantity was estimated from freshly harvested 500 g bitter gourd fruits at the edible stage in all treatment combinations. The DPPH (1, 1-diphenyl-2-trinitrobenzene hydrazine) activity of bitter gourd fruit samples was estimated as suggested by Blois (18). The chlorophyll content of fruits was estimated using the standard protocol (19). Vitamin C content was estimated as per the procedure described by Goo et al. (20). β-carotene was extracted from the powdered pericarp of the bitter gourd fruits using the protocol described in the study by Patel et al. (21) and characterized quantitatively using supercritical fluid chromatography-based ultra-performance conversance chromatography (Acquity UPC^2^ system, Waters Technologies, USA). Empower^3^ software was used to operate the system during the quantitative analysis of the samples. The total saponins were extracted in an aqueous, two-phase extraction system (22). Charantin was estimated following the method of Kim et al. (23) using an HPLC (NS-4000 model) with a UV detector at a wavelength of 204 nm.

Bitter gourd fruits were dried, ground, and digested to estimate the concentrations of K and four micronutrients: Zn, Fe, Mn, and Cu. The concentration of K was determined using a flame photometer and compared with standards ranging from 0 to 100 mg kg^−1^ of potassium chloride. An atomic absorption spectrophotometer was used to estimate the concentrations of Zn, Fe, Mn, and Cu (24). The most sensitive wavelengths were 213.7, 248.7, 279.5, and 324.6 nm for Zn, Fe, Mn, and Cu, respectively.

### 2.8 Data analyses

Averages over 3 years in each environment were compared using the least significant difference (LSD) test at a 95% confidence interval. SAS software, version 9.4, was used for the analysis of variance (ANOVA).The genotype × yield × trait (GYT) biplot approach was used to evaluate treatments across multiple traits using R software. In this approach, the genotype × trait (GT) two-way table (treatments vs. traits) was first transformed into a genotype × yield × trait (GYT) two-way table. The GYT table was then displayed in a GYT biplot. Four patterns were presented in GYT polygons: “Which, won, where/what,” “mean vs. stability,” “ranking genotypes,” and “ranking environments.” The “Which, won, where/what” polygons assist in identifying the outperforming treatment across the yield × traits and the interaction pattern between treatments × yield × traits (25). Similarly, “mean vs. stability” patterns are used to identify stable treatments across diverse variables (26, 27). The average tester coordination (ATC) view of the GYT biplots in “mean vs. stability” is used to rank treatments based on their stability across the yield-trait interactions and to illustrate their trait performances.

Similarly, “ranking genotypes” and “ranking environments” are merely 2-D graphs that arrange the treatments and yield × traits in order of performance in varied mega-environments (26, 27). To design 2-D GYT biplots, singular value data decomposition was employed, and the first two principal components (PCs) were generated. The data were centered on the yield × traits columns while comparing bitter gourd treatments and centered on the bitter gourd treatments when comparing yield × traits, for “which, won where/what” polygon patterns, symmetric scaling (f = 0.5) was applied. The angles between yield × trait vectors defined the correlations (28, 29).

## 3 Results

### 3.1 Microclimate parameters

The cladding materials influence microclimatic variables such as temperature, relative humidity, and light intensity within protected structures. The mean maximum and minimum temperatures within the protected structures gradually rose as the seasons transitioned from winter to summer (February–May 2018 and 2019). Conversely, the mean maximum and minimum temperatures within the protected structures gradually declined as the seasons transitioned from summer to winter (August–November 2020) across all structures except the high-tech greenhouse. The mean relative humidity (RH) was highest in February and August and gradually declined until the end of the cropping season, i.e., May and November. The light intensity measured as photosynthetically active radiation (PAR) reached its peak in February–March and August–September within the naturally ventilated polyhouse and insect-proof net house structures. However, from April and November onwards, PAR declined due to the implementation of mobile shade nets inside these structures to maintain lower temperatures. In the high-tech greenhouse, the temperature and relative humidity were maintained according to crop requirements throughout the growing period in both seasons. The stable temperature was maintained by operating cooling pads and fans during the summer season (March–May) and heaters and blowers during the winter season (October–November). Humidity was maintained by operating dehumidifiers and foggers.

### 3.2 Antioxidants in fruits

**Figure 2:**
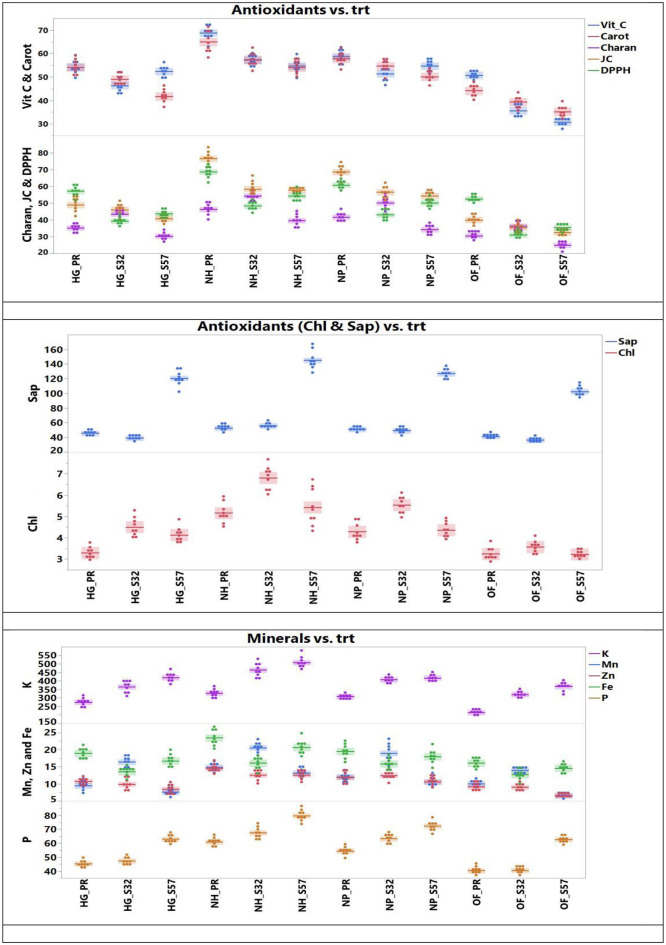
Antioxidants and mineral concentrations observed in bitter gourd fruits in different experimental treatment combinations.

The ANOVA for antioxidant traits is presented in Table 3. The concentration of antioxidants in bitter gourd fruits increased when they were grown in protected environments. The juice concentration was only 36.4, 36.0, and 35.5 ml per 500 g^−1^ fruit weight (FW) in treatments T10, T11, and T12 (open field + Pusa Rasdar, S32, and S57, respectively). This increased to 62.9, 66.6, and 63.3 per 500 g^−1^ FW in treatments T7, T8, and T9, respectively (Figure 2).

**Table 3:** ANOVA of antioxidants and dietary nutrient concentrations in the fruits of bitter gourd.

**Source of variation**	**DF**	**Juice content (ml/500 g)**	**DPPH (mg/100 g)**	**Chlorophyll (mg/100 g)**	**Vitamin C (mg/100 g)**	**Carotenoid (mg/100 g)**	**Saponin (μg/g)**	**Charantine (μg/g)**	**P (mg/100 g)**	**K (mg/100 g)**	**Mn (mg/100 g)**	**Zn (mg/100 g)**	**Fe (mg/100 g)**
Replication	2	0.85	1.05	0.97	6.28	27.58	88.10	5.30	7.13	427.6	1.58	1.53	0.67
E	11	13,994.56	4,475.61	91.91	6,551.38	5,642.72	9,245.23	3,985.14	8,153.48	250,812.1	602.4	411.24	479.2
G	2	2,904.76	7,190.32	23.96	2,503.23	1,789.64	144,001.63	3,452.18	7,269.08	416,504.8	1,240.1	76.20	445.5
G × E	22	910.56	292.78	15.16	1,204.72	369.80	3,436.59	315.76	870.82	36,200.3	124.3	71.21	135.1
Error	70	464.45	309.35	6.72	324.84	344.35	1,684.65	291.50	323.59	30,968.1	55.2	44.85	141.0
Total	107	18,275.19	12,269.10	138.72	10,590.43	8,174.09	158,456.21	8,049.89	16,624.09	734,912.9	2,023.6	605.03	1,201.5

Similarly, concentrations of DPPH increased from 39.5, 40.4, and 39.0 mg per 100 g^−1^ FW in treatments T10, T11, and T12 to 57.3, 58.0, and 55.9 mg per 100 g^−1^ FW in treatments T7, T8, and T9, respectively. Chlorophyll content also increased from 3.31, 3.42, and 3.32 mg per 100 g^−1^ FW in treatments T10, T11, and T12 to 5.52, 5.85, and 6.03 mg per 100 g^−1^ FW in treatments T7, T8, and T9, respectively. Treatments T7 (61.1 mg per 100 g FW), T8 (59.2 mg per 100 g FW), and T9 (60.4 mg per 100 g^−1^ FW) also observed higher concentrations of vitamin C compared to treatments T10 (39.1 mg per 100 g FW), T11 (38.9 mg per 100 g FW), and T12 (39.3 mg per 100 g^−1^ FW).

The protected structures significantly improved the concentrations of antioxidants such as carotenoids, saponins, and charantins in bitter gourd fruits. The lowest concentrations of carotenoids (40.5, 39.9, and 38.3 mg per 100 g^−1^ FW), saponin (58.5, 60.1, and 61.8 μg g^−1^ FW), and charantin (30.9, 30.7, and 30.1 μg g^−1^ FW) were observed in treatments T10, T11, and T12, respectively. These increased to 60.0, 57.4, and 59.0 mg per 100 g^−1^ FW in carotenoids, 85.5, 81.1, and 86.8 μg per g^−1^ FW in saponin, and 46.0, 47.3, and 46.8 μg per g^−1^ FW in charantin concentrations in treatments T7, T8, and T9, respectively (Table 4). Antioxidant concentrations were not statistically different among treatments T7, T8, and T9.

**Table 4:** Mean performance of antioxidants and dietary nutrient concentration in the fruits of bitter gourd in different environments.

**Treatment**	**Juice content (ml/500 g)**	**DPPH (mg/100 g)**	**Chlorophyll (mg/100 g)**	**Vitamin C (mg/100 g)**	**Carotenoid (mg/100 g)**	**Saponin (μg/g)**	**Charantine (μg/g)**	**P (mg/100 g)**	**K (mg/100 g)**	**Mn (mg/100 g)**	**Zn (mg/100 g)**	**Fe (mg/100 g)**
T1	42.72	47.38	3.85	51.21	47.57	64.69	36.40	52.30	348.81	10.81	9.56	15.68
T2	46.44	46.08	3.92	51.75	48.37	72.05	35.58	50.86	352.49	11.43	9.08	17.09
T3	46.72	46.62	4.16	49.98	49.02	68.44	36.94	52.24	358.34	11.17	10.43	16.44
T4	58.62	49.68	4.71	54.24	53.75	76.95	41.47	63.20	381.49	13.44	10.97	18.16
T5	60.34	51.74	4.74	55.14	54.59	74.42	42.50	63.37	378.82	13.88	11.72	17.31
T6	60.43	52.72	4.74	55.35	54.32	75.64	42.04	63.46	370.82	14.21	12.25	17.81
T7	62.94	57.29	5.52	61.08	59.99	85.55	46.02	68.17	428.75	16.25	12.51	19.16
T8	66.62	57.97	5.85	59.16	57.40	81.14	47.31	70.51	422.55	16.06	13.39	20.58
T9	63.32	55.85	6.03	60.38	58.96	86.81	46.79	69.45	448.41	16.20	13.78	20.44
T10	36.42	39.55	3.31	39.13	40.49	58.46	30.92	47.64	305.90	9.72	8.06	13.97
T11	36.04	40.41	3.42	38.88	39.92	60.09	30.70	47.95	293.35	10.39	8.11	14.69
T12	35.51	38.97	3.32	39.30	38.28	61.79	30.08	47.80	302.07	10.45	8.56	14.64
LSD (*P* = 0.05)	2.427	1.981	0.292	2.030	2.09	4.622	1.92	2.03	19.82	0.84	0.75	1.34
**Year**												
Y1	58.49	59.78	3.99	58.11	55.27	47.69	38.42	50.22	281.11	11.57	11.58	19.47
Y2	49.16	40.39	5.11	47.67	50.08	45.02	46.05	54.62	388.88	17.47	10.96	14.53
Y3	46.37	45.88	4.29	48.12	45.31	123.78	32.22	69.40	427.97	9.46	9.57	17.49
LSD (*P* = 0.05)	1.21	0.99	0.15	1.02	1.05	2.31	0.96	1.013	9.91	0.42	0.38	0.67
T × Y	4.204	3.43	0.51	3.516	3.62	8.01	3.33	3.509	34.33	1.45	1.31	2.32

A significant (*p* > 0.05) interaction effect between breeding lines and environments over the years was observed for different antioxidants. In bitter gourd fruits, the highest concentrations of juice (66.6 ml per 500 g^−1^ FW), DPPH (58.0 mg per 100 g^−1^ FW), and charantin (47.3 μg per g^−1^ FW) were observed in treatment T8, although there was no statistical difference between T8, T9, and T7. The highest concentrations of chlorophyll (6.03 mg per 100 g^−1^ FW) and saponin (86.8 μg per g FW) were observed in treatment T9, while T7 had the highest concentration of vitamin C (61.08 mg per 100g^−1^ FW) and carotenoids (60.0 mg 1 per 00 g^−1^ FW).

The mean performance (Year-I, II, and III) for the growing environment and breeding lines highlights that the maximum juice content (58.5 ml per 500 g^−1^ FW) and antioxidants such as DPPH (59.8 mg per 100 g^−1^ FW), vitamin C (58.1 mg 100 g^−1^ FW), and carotenoids (55.3 mg 100 g^−1^ per FW) were maximum in Year I. The highest chlorophyll content (5.11 mg per 100 g^−1^ FW) and charantin (46.05 μg per g^−1^ FW) were observed in Year II, whereas the highest concentration of saponin (123.78 μg per g^−1^ FW) was observed in Year III (Table 4).

### 3.3 Phosphorus, potassium, and micronutrient concentrations in fruits

The concentrations of phosphorus, potassium, and micronutrients increased significantly in different protected environments compared to open-field-grown crops. The highest concentrations of P (70.5 mg per 100 g^−1^ FW) and Fe (20.6 mg per 100 g^−1^ FW) were observed in treatment T8. The maximum contents of K (448.4 mg per 100 g^−1^ FW) and Zn (13.78 mg per 100 g^−1^ FW) were observed in treatment T9. The greatest Mn content (16.25 mg per 100 g^−1^ FW) was recorded in treatment T7. However, there were no significant differences in these nutrients among treatments T7, T8, and T9 (Figure 2).

The lowest content of P (47.6 mg per 100 g^−1^ FW), Mn (9.7 mg per 100 g^−1^ FW), Zn (8.06 mg per 100 g^−1^ FW), and Fe (13.97 mg per 100 g^−1^ FW) were detected in treatment T10. The lowest concentration of K (293.4 mg 100 g^−1^ FW) was observed in treatment T11 (Table 4). Despite these variations, no statistical differences were observed in dietary nutrient concentrations among treatments T7, T8, and T9.

A significant (*p* > 0.05) interaction between breeding lines and environments over the years was found for different dietary nutrient concentrations. The mean performance (Years I, II, and III) of the growing environment and breeding lines highlighted that mineral nutrients such as P (69.40 mg 100 g^−1^ FW) and K (427.97 mg 100 g^−1^ FW) reached their maximum concentrations in Year III. The highest Mn concentration (17.47 mg per 100 g^−1^ FW) was recorded in Year II, whereas the maxima for Zn (11.58 mg per 100 g^−1^ FW) and Fe (19.47 mg per 100 g^−1^ FW) were noted in Year I (Table 4).

### 3.4 Earliness and yield traits

**Figure 3:**
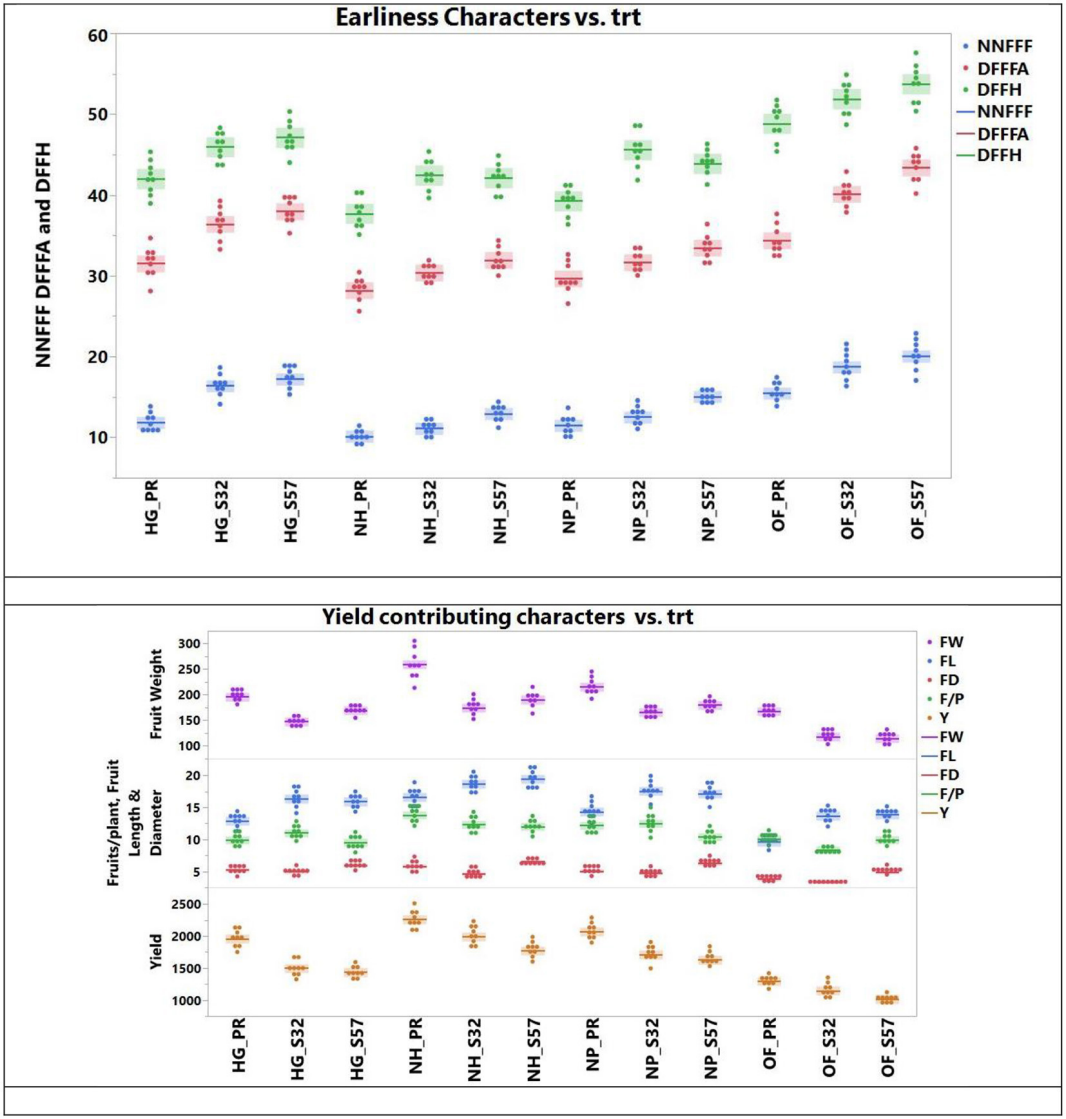
Earliness and yield traits observed in bitter gourd lines in different experimental treatment combinations (T8 > T7 > T9 were superior for earliness and yield traits).

The ANOVA for earliness and yield traits is given in (Table 5). Among the diverse growing environments and treatment interactions, treatment T9 (S57 + net house) produced the lowest node number of first female flowers (11.20), which remained on par with treatment T8 (S32 + net house) and treatment T7 (Pusa Rasdar + net house). Treatment T7 required the fewest days for the first female flower anthesis (29.2), which was statistically similar to treatments T8 and T9. For days prior to the first fruit harvest, treatment T9 had the shortest duration (39.7), which was on par with treatments T7 and T8 (Figure 3). The greatest fruit length (18.5 cm) was observed with T9, which was on par with treatments T8 (18.3 cm) and T7 (18.0 cm). Furthermore, the highest fruit diameter was recorded in treatment T8 (5.86 cm), followed by treatment T7 (5.68 cm).

**Table 5:** ANOVA for earliness and yield traits in bitter gourd.

**Source of variation**	**DF**	**NNFFF**	**DFFFA**	**DFFH**	**Fruit length (cm)**	**Fruit diameter (cm)**	**Average fruit weight (g)**	**Number of fruits/plants**	**Yield/plant (g)**
		**SS**	**SS**						
Replication	2	3.3	3.2	2.64	1.23	0.26	108.1	2.05	15,303.9
E	11	692.2	1,386.8	1,802.6	499.8	45.21	80,172.5	189.15	10,890,673.6
G	2	304.5	611.6	517.3	248.7	39.11	68,204.8	18.17	3,612,950.6
G X E	22	68.0	134.9	100.9	38.6	8.35	13,518.4	68.15	485,580.8
Error	70	79.4	160.0	222.9	71.9	11.53	9,354.7	52.81	754,857.7
Total	107	1,147.3	2,296.6	2,646.5	860.2	104.45	171,358.4	330.29	15,759,366.7

NNFFF, Node number of first female flower; DFFFA, Days until first female flower anthesis; DFFH, Days until first fruit harvest.

The highest average fruit weight was recorded in treatment T9 (210.1 g), which was on par with treatment T8 (209.4). Treatment T8 was superior in the number of fruits per plant (13.1), followed by treatments T9 (12.7) and T7 (12.4). Treatment T9 was the top performer for yield per plant (2,060 g), followed by treatments T8 (2,000.4 g) and T7 (1,967.7 g; Table 6).

**Table 6:** Mean performance of earliness and yield traits in different environments in bitter gourd fruits.

**Treatment combination**	**NNFFF**	**DFFFA**	**DFFH**	**Fruit length (cm)**	**Fruit diameter (cm)**	**Average fruit weight (g)**	**Fruits/plant**	**Yield/plant (g)**
HG_PR	14.96	36.05	45.81	14.83	5.53	166.41	9.77	1,612.98
HG_S32	15.23	34.33	44.14	15.21	5.16	171.66	10.08	1,659.44
HG_S57	15.23	35.52	45.15	15.22	5.44	175.40	10.63	1,625.13
NP_PR	13.42	32.33	42.29	16.83	5.24	184.78	11.26	1,854.13
NP_S32	12.42	31.50	42.57	16.48	5.42	185.55	12.04	1,764.29
NP_S57	13.23	30.97	43.89	15.82	5.42	191.21	11.83	1,795.33
NH_PR	11.67	29.24	41.24	18.04	5.68	203.36	12.41	1,967.73
NH_S32	11.33	30.59	41.38	18.30	5.86	209.35	13.08	2,000.37
NH_S57	11.20	30.72	39.67	18.46	5.51	210.11	12.72	2,059.99
OF_PR	18.82	39.65	52.32	12.77	4.06	132.13	9.09	1,109.98
OF_S32	18.15	39.04	51.86	12.55	4.12	134.37	9.59	1,152.46
OF_S57	17.27	39.20	50.31	11.91	3.94	134.41	9.66	1,201.47
LSD (*P* = 0.05)	1.00	1.43	1.68	0.95	0.38	10.89	0.82	97.84
**Years**								
Y1	12.23	30.94	41.96	13.39	4.99	209.78	11.49	1,898.67
Y2	14.68	34.64	46.47	16.59	4.45	151.56	11.05	1,588.54
Y3	16.32	36.70	46.73	16.62	5.91	163.35	10.49	1,463.61
LSD (*P* = 0.05)	0.50	0.71	0.84	0.48	0.19	5.45	0.41	48.92
T × Y	1.74	2.467	NS	1.65	0.66	18.87	1.42	169.47

NNFFF, Node number of first female flower; DFFFA, Days until the first female flower anthesis; DFFH, Days until the first fruit harvest.

The mean performance (Years I, II, and III) of different growing environments and breeding lines highlights that the earliness characteristics, such as the lowest node number of the first female flower, minimum days to the first female flower anthesis, and minimum days to the first fruit harvest, were observed in Year I. The maximum fruit length and fruit diameter were recorded in Year II, although there was no statistical difference between Year II and Year III. Some yield parameters, such as average fruit weight, the number of fruits per plant and yield per plant, and the mean of Year I, showed superior performance. However, Year II showed poor earliness and yield trait performance compared to Years I and III.

### 3.5 GYT biplot analysis

The GYT biplot analysis was conducted on juice content and antioxidants (such as DPPH, chlorophyll, vitamin C, carotenoids, saponin, and charantin), as well as dietary nutrients (P, K, Mn, Zn, and Fe) in bitter gourd fruits. In the GYT analysis, 12 treatment combination effects involving four raising environments and three breeding lines/cultivars of bitter gourd were examined.

#### 3.5.1 GYT biplot for antioxidants in bitter gourd fruits

**Figure 4:**
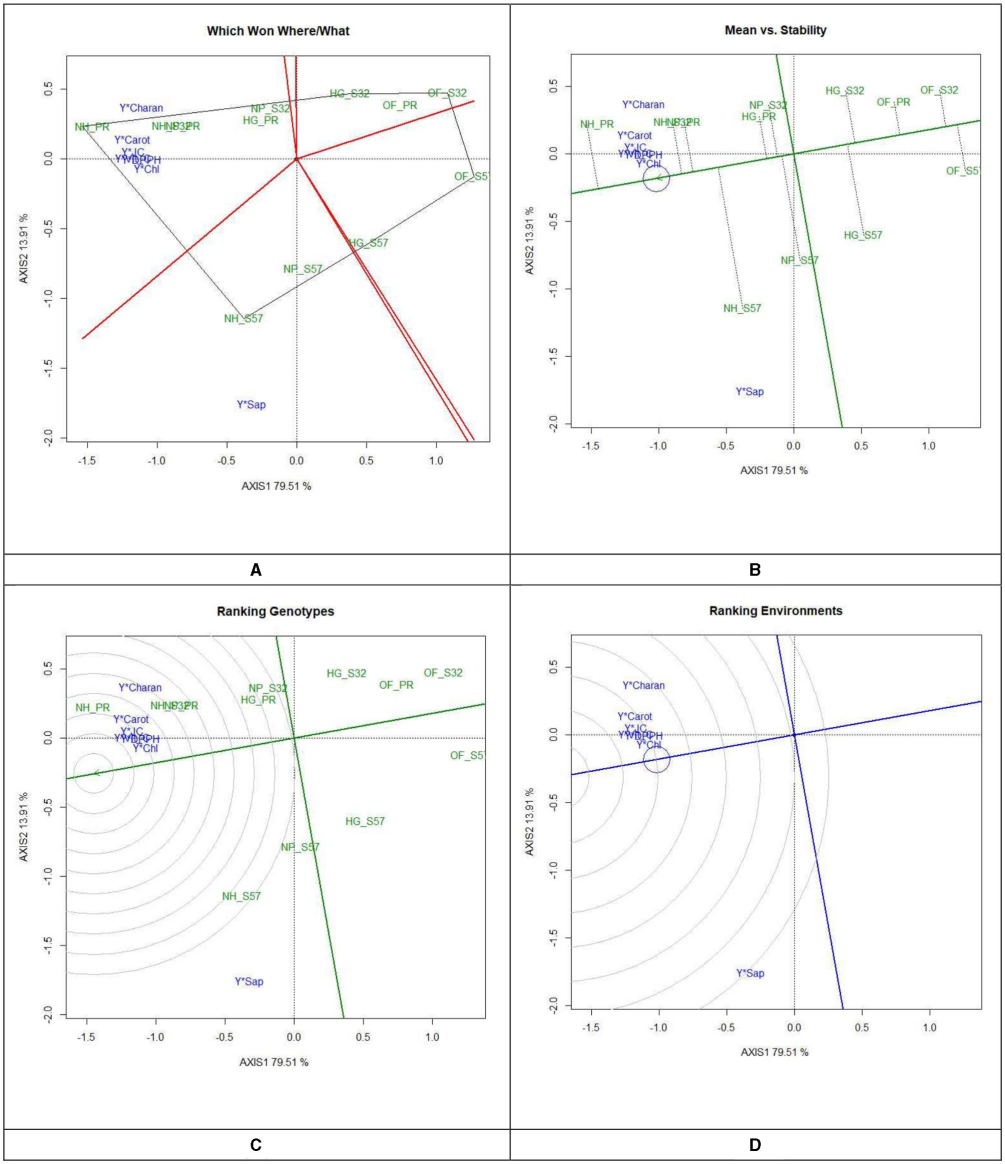
GYT biplot analysis of antioxidants in bitter gourd fruits in experimental treatments **(A)** polygon view (which won where/what), **(B)** mean vs. stability, **(C)** ranking genotypes (mean ranking treatments), and **(D)** ranking environments. AXIS 1: principal component 1, AXIS 2: principal component 2. #1= HG_PR; 2= HG_S32; 3= HG_S57; 4= NP_PR; 5= NP_S32; 6= NP_S57; 7= NH_PR; 8= NH_S32; 9= NH_S57; 10= OF_PR; 11= OF_S32; 12= OF_S57.

In the GYT biplot analysis of yield by antioxidants, the first two principal components (PC) explained 79.5 and 13.9% of the variation, respectively. The 2-D polygon showed that S32, when grown in a net house, generated the highest yield by juice (66.6 ml per 500 g^−1^ FW), yield by charantin (47.3 μg per g^−1^ FW), and yield by DPPH (58.0 mg per 100 g^−1^ FW). In contrast, Pusa Rasdar, when raised in a net house, produced the highest yield of vitamin C (61.1 mg 100 per g^−1^ FW) and carotenoids (60.0 mg per 100 g^−1^ FW). Furthermore, S57, when cultivated in net houses, produced the highest yield of saponins (86.8 μg per 100 g^−1^ FW). Treatments T7, T8, and T9 were positioned in the same megaenvironment and were relatively close to each other (Figure 4). This suggests that these combinations produced the highest concentration of antioxidants in bitter gourd fruits.

After clustering all the antioxidant treatments into three convex hulls, the open-field-raised bitter gourd crops (T10, T11, and T12) performed weakly and fell into a single-mega environment. This highlights that the cultivation of improved breeding lines alone is not sufficient to enhance the antioxidant properties of bitter gourd fruits without proper crop management and growing environments. The treatment combinations T10, T11, and T12 were located in another mega-environment, implying that these three environments performed similarly. The positioning of all antioxidants in the same mega-environments indicated that, while there may be significant variations between the growing environments, there were no extreme differences in the pattern of antioxidant concentrations.

#### 3.5.2 The mean vs. stability

The average environment coordinate (AEC) of a biplot is utilized to ascertain antioxidant concentrations and enhance treatment stability. The AEC ordinates represent the normal lines intersecting the origin of the biplot (Figure 4B). The points distant from the origin indicate lower stability and higher performance of treatment × yield × trait interactions in both directions along the AEC ordinate. The instability of a treatment on the AEC directly correlates with the absolute length of its projection. Treatments T7, T8, and T9 were identified as the most stable treatments concerning antioxidant concentration and stability.

#### 3.5.3 Ranking experimental treatments

In GYT biplots, treatments are ranked to establish the efficiency order of the treatments (Figure 4C). The treatment combination that yields the highest antioxidant concentration and greater stability across the tested environments is regarded as the most effective treatment; this is highlighted with concentric circles. The treatments identified as 'ideal' are positioned closer to the concentric circles, exhibiting elevated mean antioxidants and greater stability in antioxidant concentrations. Of the 12 treatment combinations, treatment T7 was identified as the best treatment in the inner orbit, followed by treatments T8 and T9 in the second orbit. Treatments T12 > T11 > T10 were outside the circle and showed their relatively lower performance with respect to antioxidants in bitter gourd fruits. Figure 4C ranks the antioxidant concentrations relative to the “model environment,” depicted by the smallest circle on the AEC axis. An antioxidant positioned near the intersection of the straight lines holds a superior ranking, while those farther from the intersection are ranked lower.

#### 3.5.4 Ranking yield by antioxidants

The yield by antioxidant concentration in bitter gourd fruits was observed in the order of yield × chlorophyll > yield × DPPH > yield × vitamin-C > yield × carotenoids > yield × charantin > yield × saponin (Figure 4D). In Figure 4D, yield × chlorophyll and yield × DPPH are closer to the circle, indicating higher efficiency and stability, while the other antioxidants are farther away, suggesting lower performance relative to the “model environment.”

### 3.6 GYT biplot for dietary nutrients

**Figure 5:**
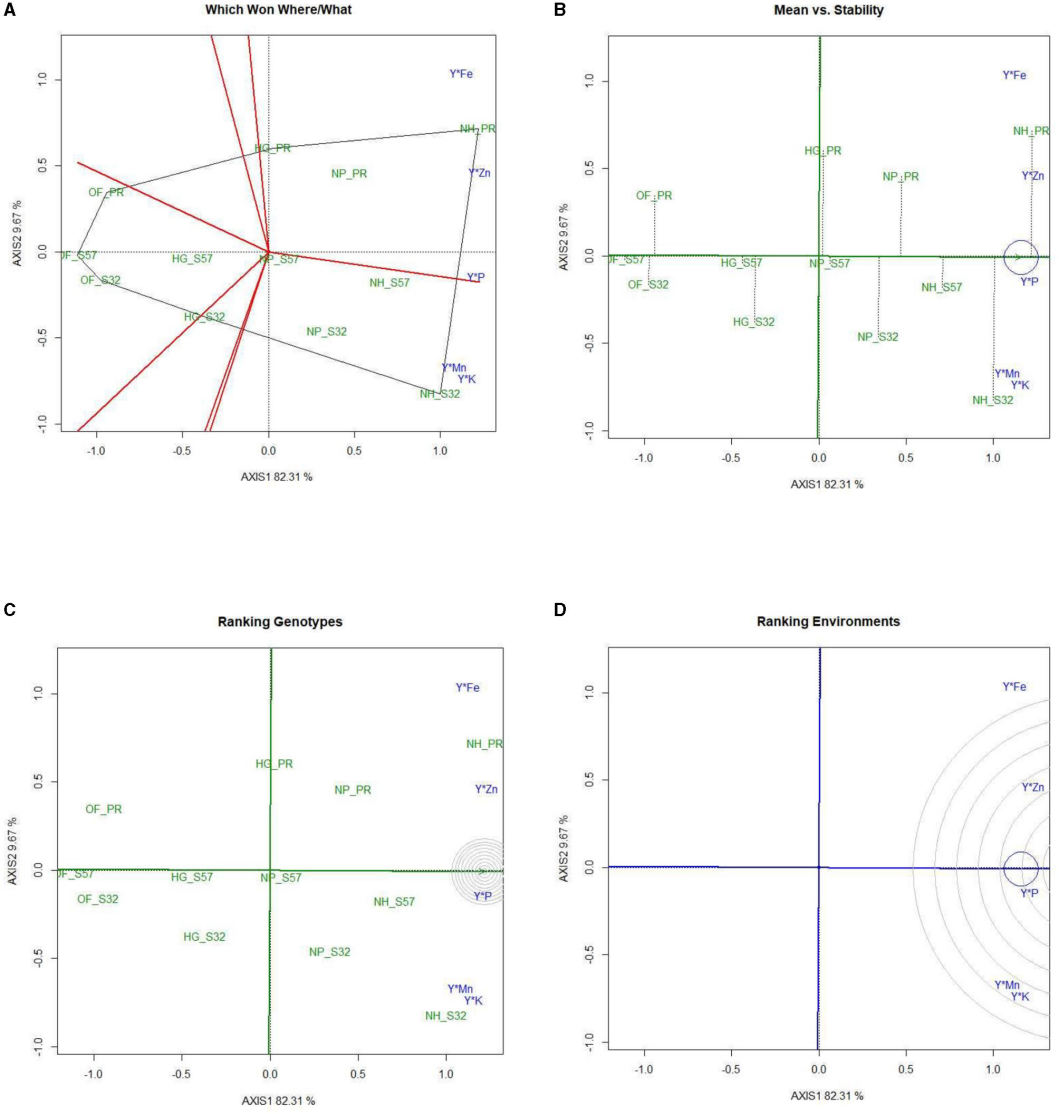
GYT biplot analysis of mineral nutrient concentrations in bitter gourd fruits in experimental treatments **(A)** polygon view (which won where/what), **(B)** mean vs. stability, **(C)** ranking genotypes (mean ranking treatments), and **(D)** ranking environments. AXIS 1: principal component 1, AXIS 2: principal component 2. #1= HG_PR; 2= HG_S32; 3= HG_S57; 4= NP_PR; 5= NP_S32; 6= NP_S57; 7= NH_PR; 8= NH_S32; 9= NH_S57; 10= OF_PR; 11= OF_S32; 12= OF_S57.

In the GYT biplot analysis of yield × mineral nutrients in bitter gourd fruits, the first two PCs elucidated 82.3 and 9.7% of the variation, respectively. The “which, won where/what” 2-D plot indicated that treatment T8 had outperformed for K and Mn concentrations. Similarly, T7 was superior for Zn and Fe and T9 for P, as these were observed to be the best treatments (Figure 5A). The presence of all mineral nutrients in the same mega-environments establishes that all these parameters follow a uniform pattern of performance in the given set of treatments. Treatments T4, T5, and T6 maintained their position as the second most effective group of treatments with regard to dietary nutrient concentrations. The treatments conducted in open-field conditions (T10, T11, and T12) demonstrated subpar performance with respect to mineral nutrient levels, as illustrated by their presence in a single mega-environment.

The mean *vs*. stability biplots revealed that treatments T8, T7, and T9 stood out as optimal choices, exhibiting both high mineral nutrient content and stability (Figure 5B). The ranking treatment graph displayed the performance hierarchy of different experimental treatments, indicating that for mineral nutrients, the order was T8 > T7 and T9. Notably, all these treatments were grouped within the same convex hull, as depicted in Figure 5C. Treatments T12 > T11 > T10 performed relatively suboptimally for mineral nutrients. The ranking environment polygon illustrated that P exhibited the highest stability, followed by Zn. K and Mn were located within the same orbit, which signifies their greatest stability levels (Figure 5D). Fe was farthest from the concentric circle, indicating that it was the least stable mineral nutrient.

### 3.7 GYT biplot for earliness and yield traits

**Figure 6:**
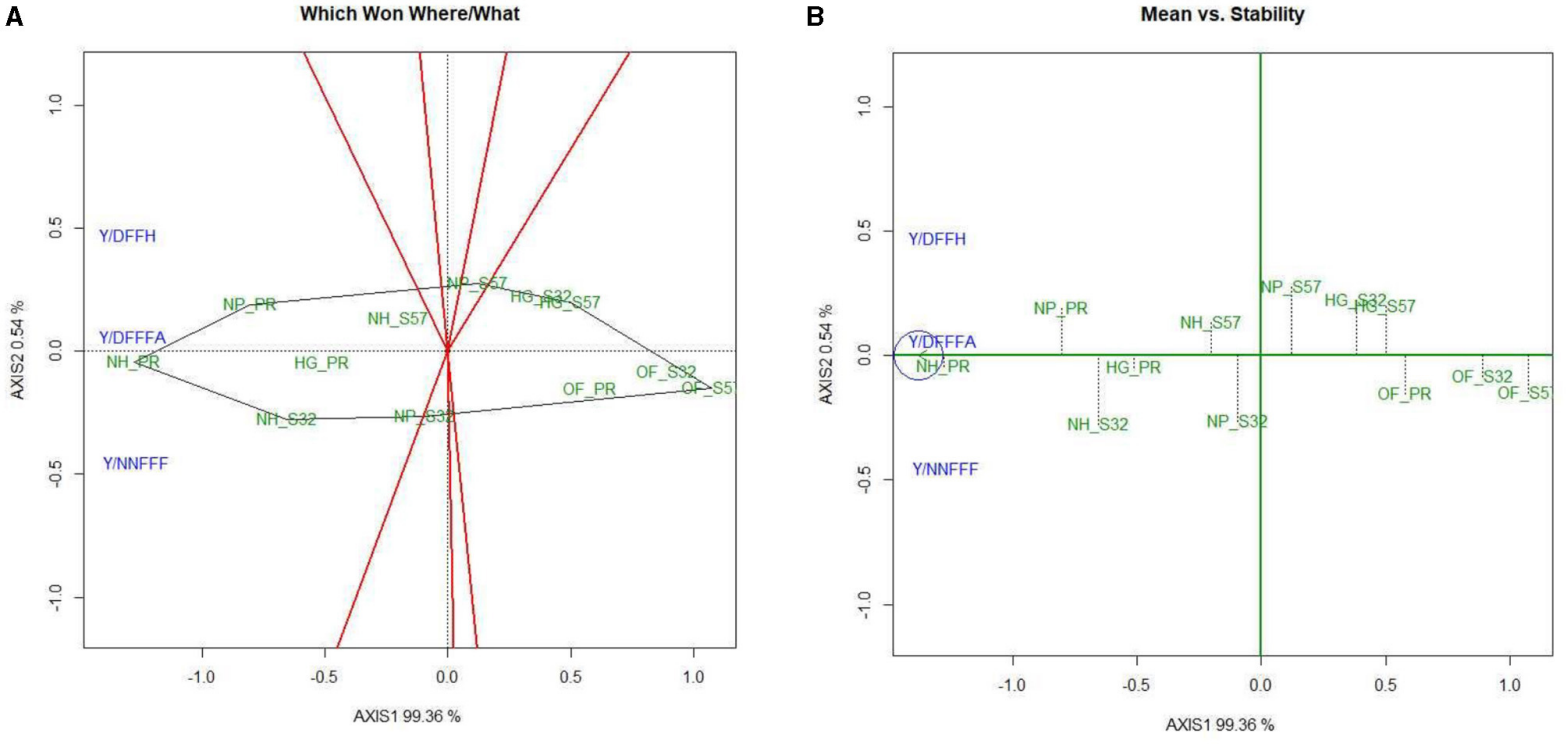
GYT biplot analysis of earliness traits in bitter gourd fruits in experimental treatments **(A)** polygon view (which won where/what) and **(B)** mean vs. stability. AXIS 1: principal component 1, AXIS 2: principal component 2. #1= HG_PR; 2= HG_S32; 3= HG_S57; 4= NP_PR; 5= NP_S32; 6= NP_S57; 7= NH_PR; 8= NH_S32; 9= NH_S57; 10= OF_PR; 11= OF_S32; 12= OF_S57.

**Figure 7:**
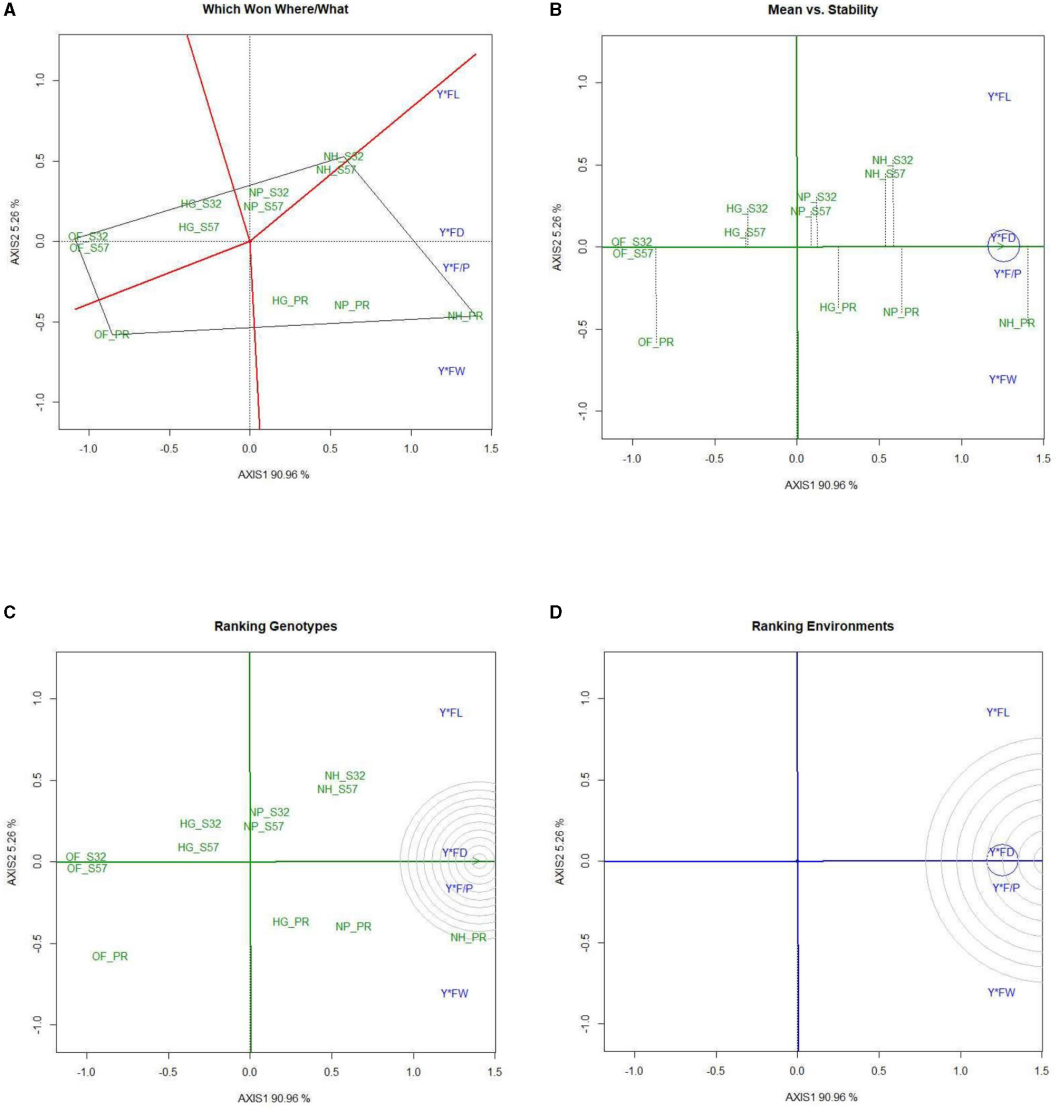
GYT biplot analysis of yield traits in bitter gourd fruits in experimental treatments **(A)** polygon view (which won where/what), **(B)** mean vs. stability, **(C)** ranking genotypes (mean ranking treatments), and **(D)** ranking environments. AXIS 1: principal component 1, AXIS 2: principal component 2. #1= HG_PR; 2= HG_S32; 3= HG_S57; 4= NP_PR; 5= NP_S32; 6= NP_S57; 7= NH_PR; 8= NH_S32; 9= NH_S57; 10= OF_PR; 11= OF_S32; 12= OF_S57.

A GYT analysis for earliness and yield traits in terms of their performance in the experimental treatments was also undertaken (Figures 6, 7). The first PC explained 99.36 and 0.54% of the variation, respectively, in the GYT biplots of yield × earliness traits in bitter gourd fruits. Similarly, 90.96 and 5.26% of the variation were observed for the first two PCs, respectively, in the GYT biplots of yield traits in bitter gourd fruits. The “which, won where/what” polygon displayed that in terms of earliness traits, such as days until the first female flower anthesis, the node number of the first female flower, and days until the first fruit harvest, treatment T7 exhibited the highest performance, followed by treatments T4 and T8 (Figure 6A). The order of treatments (T8 > T7 > T9) demonstrated superior performance concerning yield and its associated traits.

The mean *vs*. stability biplots suggested that treatments T7, followed by T4, T8, and T9, had the highest stability for earliness and yield-associated traits. However, days until the first female flower anthesis and fruit yield per plant were the most stable traits among different treatment combinations (Figure 6B). In Figure 6, it is evident that within earliness traits, the stability was the lowest for the node number of the first female flower and days to the first fruit harvest. Similarly, fruit length and fruit weight exhibited the least stability.

The “ranking treatment” biplots presented the performance sequence of different experimental treatments. The order for yield traits was T7 > T8, and T9. Conversely, treatments T11 > T12 > T10 demonstrated comparatively inferior performance for both earliness and yield traits (Figure 7).

The “ranking environments” polygons highlighted that fruit yield per plant was the most stable trait, followed by fruit diameter and the number of fruits per plant. Fruit weight and fruit length were farthest from the concentric circle and were the least stable traits (Figure 7).

### 3.8 Interrelationships between different studied characters

**Figure 8:**
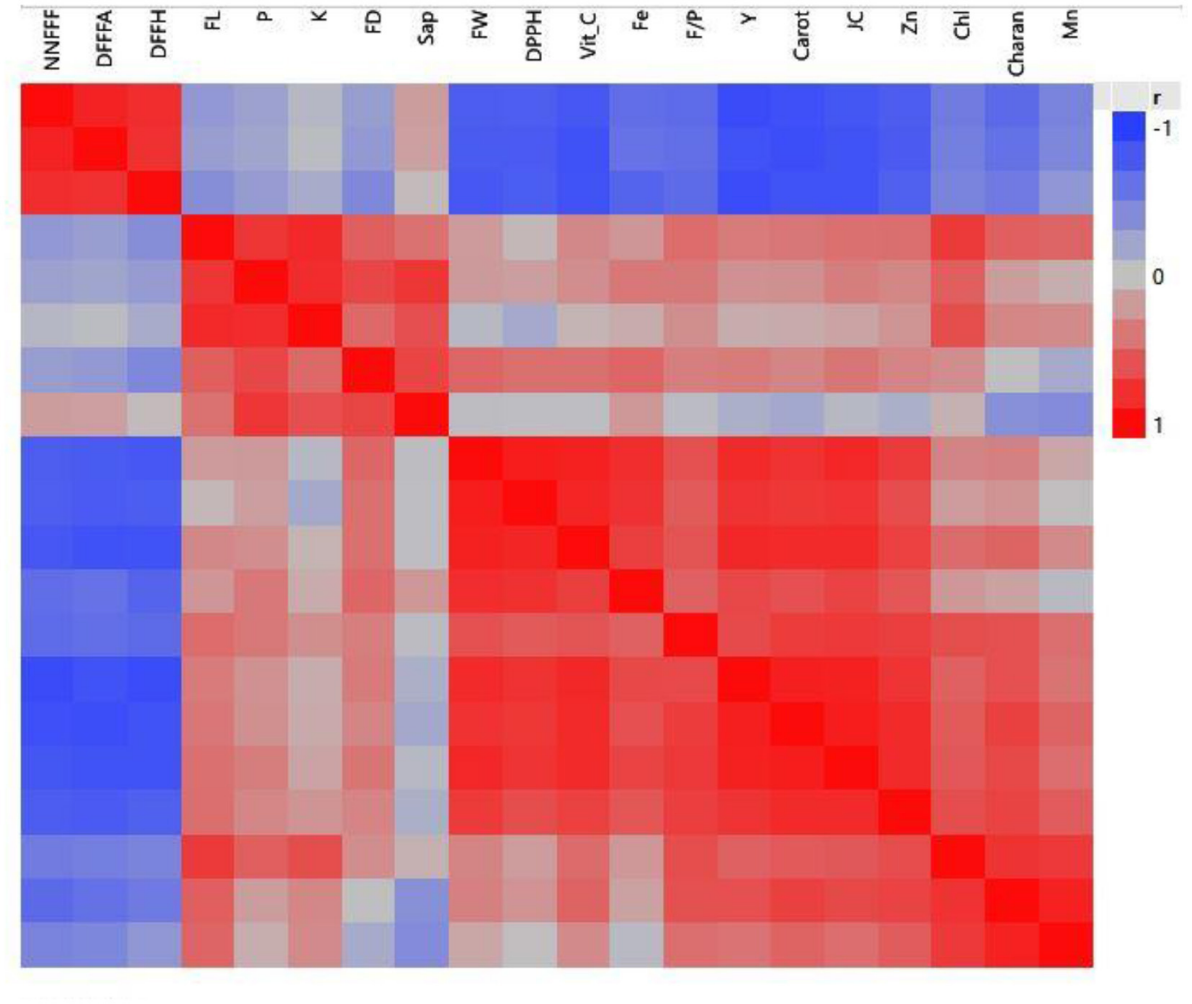
A correlation study of characters in the bitter gourd grouped these characters into four groups.

Initially, the interrelationship among earliness, yield-contributing traits, and mineral and antioxidant characteristics was studied using various statistical tests. The correlation analysis (Figure 8) clearly grouped the characters into four groups: group I (NNFFF, DFFFA, and DFFH), group II (FL, FD, F/P, K, Saponin, FW, DPPH, Vitamin C, Fe, and P), group III (yield, juice content, and Zn), and group IV (chlorophyll, charantine, and Mn).

### 3.9 Regressions of yield character on mineral, antioxidant, earliness, and yield contributing traits

**Figure 9:**
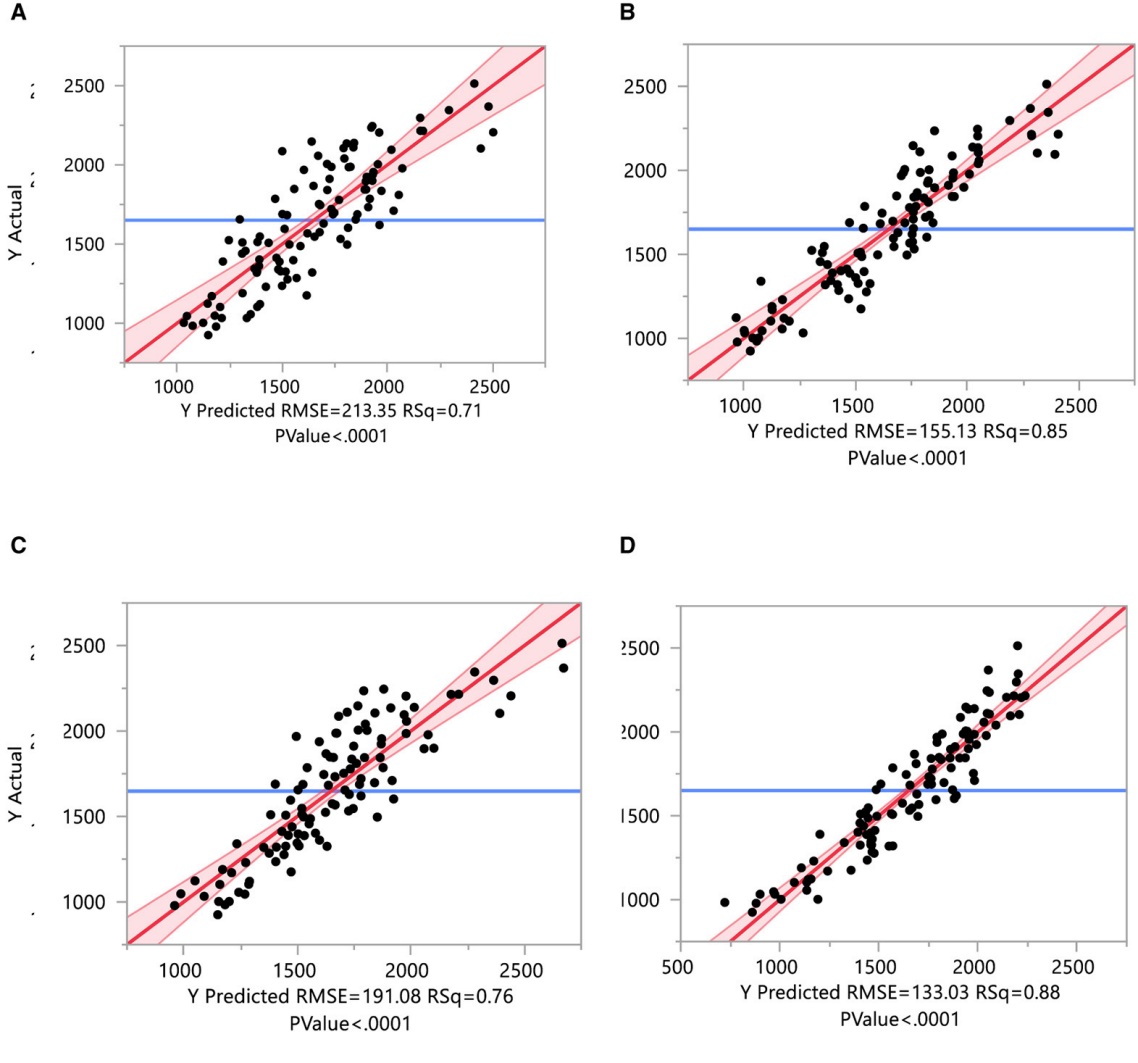
Regressions of yield parameters on mineral, antioxidant, earliness, and yield-contributing traits. These yield characters are regressed on different parameters and are grouped as yield, earliness, minerals, and antioxidant characters. **(A)** Regressions of yield on mineral traits. **(B)** Regressions of yield on antioxidant traits. **(C)** Regressions of yield on yield-contributing traits. **(D)** Regressions of yield on earliness traits.

The yield character is regressed on different parameters, which are grouped into four categories: yield traits (FL, FD, FW, and F/P), earliness traits (NNFFF, DFFFA, and DFFH), minerals (P, K, Mn, Zn, and Fe), and antioxidant characters (juice content, DPPH, chlorophyll, vitamin C, carotenoids, saponin, and charantin). When yield is regressed on yield-contributing traits (*R*^2^ = 0.76), all traits significantly contribute to yield, indicating that yield is directly affected by these characters (Figure 9 and Supplementary Table 2). When yield is regressed on earliness traits (*R*^2^ = 0.88), all earliness traits are found to negatively and significantly contribute to yield (Figure 9 and Supplementary Table 2). Mn, Zn, and Fe significantly contribute to yield when regressing yield on minerals (*R*^2^ = 0.71; Figure 9 and Supplementary Table 2). Only juice content and carotenoid significantly contribute to yield when regressing yield on antioxidants (*R*^2^ = 0.85; Figure 9 and Supplementary Table 2).

### 3.10 PCA regression of yield on different traits

**Figure 10:**
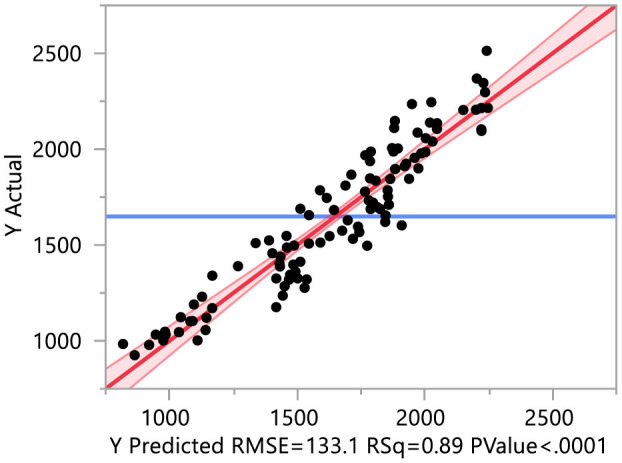
PCA regression of yield on different traits. The yield is regressed on the first principal component of antioxidants, earliness, mineral, and yield-contributing traits.

For the principal component regression, principal component analysis (PCA) was conducted on antioxidants, earliness, mineral, and yield-contributing traits. The first principal component of earliness, minerals, antioxidants, and yield-contributing traits explained 88.20, 47.87, 61.15, and 58.30% of the total variability for respective traits (Supplementary Tables 3–6). Yield is regressed on the first principal component of antioxidants, earliness, mineral, and yield-contributing traits (*R*^2^ = 0.89; Figure 10 and Supplementary Table 7). The regression results indicate that the first principal component of earliness and yield-contributing traits significantly contribute to yield. The coefficient of the first principal component of earliness is negative, while the first principal component of yield-contributing traits is positive, implying that earliness traits negatively contribute to yield while yield-contributing traits positively contribute to yield.

## 4 Discussion

The appropriate management and cultivation of bitter gourd crops in favorable environmental conditions are vital to increasing earliness, yield, fruit quality, antioxidants, and mineral nutrient contents (12, 30–32). In the present experiment, due to favorable environmental scenarios and ideal crop management throughout the growing period, antioxidants, dietary nutrient concentrations, earliness, and yield traits were significantly enhanced.

Sunlight is an important natural resource for plant growth and development, where irradiance affects plant biochemical composition and morphology. The growth of crops in polyhouses depends on light intensity, which varies from season to season. Low light intensity may cause a significant decline in photochemical activity (33). Modern greenhouse covering materials convert direct sunlight into diffuse light, which is beneficial for greenhouse crops (34). Diffused light penetrates deeper into the canopy, allowing the middle leaves to intercept more light, leading to increased photosynthesis and, thus, higher fruit production (35, 36). In this experiment, the high light intensity inside the protected structures was lower than in the open field, which was also observed in previous studies (37).

High light intensity slows down chlorophyll synthesis. Similarly, in the present experiment, high light intensity reduced chlorophyll content in open-field-grown bitter gourd varieties compared to those in protected environments. The significant increase in chlorophyll, other dietary nutrients, and antioxidants in protected structures might be due to optimal light intensity. The high yield obtained in protected structures is due to optimal light intensity and an equal distribution of radiation over the crop canopy, which results in the production of maximum photoassimilates. Optimal light intensity also leads to optimal stomatal functioning (38).

In protected structures, the cultivation of crops using drip irrigation systems coupled with mulching not only eradicates weeds but also maintains moisture in the rhizosphere for a relatively longer duration by minimizing evaporative losses, which enhances crop yield as well as nutrient biofortification (9, 32, 39, 40). The enhanced performance in terms of earliness, yield, and quality traits of the crop grown in a net house could be attributed to minimal infestation by fruit flies, white flies, leaf miners, and viral diseases. Additionally, adequate ventilation in the net house further contributed to optimal crop growth and development (41, 42). Moreover, the ventilation system in naturally ventilated polyhouses is designed to provide congenial climatic conditions for better yield performance (41). Relatively low air and soil temperatures in conjunction with high relative humidity and optimal radiation in naturally ventilated polyhouses and insect-proof net houses led to better plant growth and physiological activities, which resulted in higher yields and superior performance for earliness traits in identical agro-ecologies (43).

In the plains of North India, the growing season for cucurbits in open-field cultivation typically commences from mid-February to mid-March. However, the abrupt rise in temperatures during April and May, coinciding with the reproductive stage, leads to a decrease in female flower production, reduced fruit set, and ultimately a significant decline in yield (44). In addition to summer cultivation, cucurbits are planted during the monsoon season (which starts from mid-July to mid-August) in several parts of South Asia. However, heavy infestation of insect pests (fruit flies, red pumpkin beetles, and white flies) and diseases (leaf curl virus, mosaic virus, and yellows virus) during the wet season causes yield losses of up to 80% in open field cultivation (41). Therefore, growing bitter gourd crops in protected structures is the suggested pathway, as it diminishes diverse biotic and abiotic stressors, thereby improving the yield and quality of bitter gourd fruits (13), as also observed in the present experiment.

The supply of macronutrients such as N, P, and K in optimal proportions is crucial for plant growth and the development of an effective rooting system (45). The greater nutrient-use efficiency resulting from the application of water-soluble fertilizers through a drip irrigation system in protected structures leads to higher yields and nutrient enrichment in the edible parts of the plant. In contrast, the broadcast application of fertilizers in the hill and channel system in open field conditions for bitter gourd crops leads to heavy nutrient losses, resulting in qualitative and quantitative suboptimal performances (17, 27, 32, 46).

In the present experiment, growing bitter gourd crops in different protected structures such as naturally ventilated polyhouses, high-tech greenhouses, and insect-proof net houses, combined with improved bitter gourd breeding lines, increased the earliness traits, total yield, antioxidants, and micronutrient concentration in bitter gourd fruits. The varietal performance differs significantly with different management practices for the protected cultivation of bitter gourd fruits, particularly in nutrient accumulation such as Ca, Fe, and Zn (10). The cultivation of cv. Pusa Rasdar in insect-proof net houses was found to be better for plant growth, yield, and fruit quality traits (10). Different growing conditions had a significant influence on fruit quality traits. The maximum ascorbic acid and capsaicin content were recorded in polyhouse-grown capsicum crops compared to open fields (47). The fruit quality traits, such as total soluble solids, fruit dry matter, and lycopene content in tomatoes, were higher in naturally ventilated polyhouses, followed by insect-proof net house-grown crops (48). This increase in quality parameters in protected structures is due to modified microclimate (48).

The increase in antioxidant concentration (DPPH, chlorophyll, vitamin C, carotenoids, and saponin) and dietary nutrients (P, K, Mn, Zn, and Fe) in bitter gourd fruits may be the result of the development of improved source-sink channels, a result of more favorable environmental conditions (Table 1), improved plant growth (Figure 1), and higher nutrient accumulation due to the efficient use of fertilizers in protected structures. Growing crops in these protected environments enhances both aboveground and belowground growth of the plants (17), thereby increasing earliness, yield, and fruit quality (9, 17). The rise in antioxidants and dietary nutrients may be due to the modification of the microclimate, which reduces photosynthetically active radiation and air temperature while increasing relative humidity (44). This creates a favorable environment for crop growth, development, and physiological functioning of bitter gourd fruits inside these structures compared to open field conditions.

Improvements in growth, earliness, and yield traits were also observed in the bitter gourd fruits grown in various protected structures (10, 17, 31). Seed yield and quality, both immediately after harvest and even after 8 months of storage, were significantly superior in crops grown in insect-proof net houses compared to those grown in open fields (41). Naturally ventilated polyhouses can be recommended as the best low-tech protected structure, which modifies the microclimate to favor successful cultivation for cucumber production in the hot, arid regions of India (44). Improvement in several quality traits was also observed in the bitter gourd fruits grown in protected conditions (17, 32). Kumar et al. (49) highlighted the importance of protected structures for enhancing earliness, yield, and quality traits in cucumbers.

## 5 Conclusion

The present research has demonstrated that the bitter gourd crops cultivated in protected structures (net houses and naturally ventilated polyhouses) produce higher yields, early flowering, and enhanced concentrations of antioxidants and dietary nutrients in the fruits. Growing bitter gourd breeding lines such as S32, followed by Pusa Rasdar and S57, in net houses and naturally ventilated polyhousse is a cost-effective and sustainable strategy.

The technology is readily accessible and beneficial to farmers, providing greater yields, enriched antioxidants, and mineral nutrient biofortification in bitter gourd fruits. Earliness in protected structures also facilitates lucrative market prices in the early season. Therefore, these bitter gourd lines and their cultivation should be recommended to resource-poor farmers using low-cost protected structures such as net houses or naturally ventilated polyhouses in bitter gourd-growing regions. Even though the pollination process was carried out manually, which may increase the cost of cultivation, this cost can be compensated by earliness and high yield potential in protected structures. Future research should focus on standardizing pollination techniques using different bee species, particularly stingless bees, in different protected environments to reduce manual pollination costs and supplement farm income through honey production.

## Data availability statement

The original contributions presented in the study are included in the article/Supplementary material, further inquiries can be directed to the corresponding authors.

## Author contributions

GJ: Data curation, Investigation, Writing – original draft, Writing – review & editing. TB: Funding acquisition, Project administration, Writing – review & editing. AS: Conceptualization, Data curation, Writing – review & editing. RB: Conceptualization, Writing – original draft, Writing – review & editing. DS: Formal analysis, Writing – review & editing. SG: Formal analysis, Writing – review & editing. UR: Writing – review & editing. PR: Writing – review & editing. HR: Writing – review & editing. NV: Writing – review & editing. SK: Data curation, Writing – review & editing. BT: Supervision, Writing – review & editing.
