# Supplementary material for: Antioxidant activities, dietary nutrients, and yield potential of bitter gourd (Momordica charantia L.) lines in diverse growing environments
Source: Front Nutr. 2024 Aug 6;11:1393476. doi: 10.3389/fnut.2024.1393476 (PMC11333371; doi:10.3389/fnut.2024.1393476)
Supplement: Supplementary file 1 [file Table_1.docx]

**Table 1.** Standard meteorological weekly weather data recorded during bitter gourd cropping period in the year 2018, 2019 and 2020

| **Year** | **SMW** | **Dates** | **OF** | | | | | **IPNH** | | | | **NVPH** | | | | **HTGH** | | | |
| --- | --- | --- | --- | --- | --- | --- | --- | --- | --- | --- | --- | --- | --- | --- | --- | --- | --- | --- | --- |
|  |  |  | **Max**  **Temp**  **(°C)** | **Mini**  **Temp**  **(°C)** | **RH**  **Mean**  **(%)** | **BSS**  **(hr)** | **LI**  **(µmol.m^-2^s^-1^)** | **Max**  **Temp**  **(°C)** | **Mini**  **Temp**  **(°C)** | **RH**  **Mean**  **(%)** | **LI**  **(µmol.m^-2^s^-1^)** | **Max**  **Temp**  **(°C)** | **Mini**  **Temp**  **(°C)** | **RH**  **Mean**  **(%)** | **LI**  **(µmol.m^-2^s^-1^)** | **Max**  **Temp**  **(°C)** | **Mini**  **Temp**  **(°C)** | **RH**  **Mean**  **(%)** | **LI**  **(µmol.m^-2^s^-1^)** |
| 2018 | 6 | 05 Feb-11 Feb | 22.5 | 4.6 | 60.7 | 5.7 | 798.65 | 21.6 | 4.8 | 64.8 | 675.32 | 24.6 | 5.6 | 66.2 | 687.45 | 26.4 | 18.6 | 69.5 | 698.232 |
|  | 7 | 12 Feb-18 Feb | 23.0 | 9.2 | 69.0 | 5.9 | 765.48 | 22.4 | 9.6 | 72.2 | 652.38 | 25.7 | 11.6 | 71.6 | 663.52 | 26.6 | 18.2 | 68.8 | 683.14 |
|  | 8 | 19 Feb-25 Feb | 28.4 | 10.5 | 64.1 | 6.3 | 789.45 | 27.2 | 10.9 | 69.8 | 661.49 | 30.6 | 12.4 | 73.1 | 673.54 | 27.6 | 19.6 | 68.1 | 684.28 |
|  | 9 | 26 Feb-04 Mar | 29.0 | 12.8 | 67.6 | 6.7 | 796.32 | 28.6 | 13.1 | 73.5 | 681.47 | 31.2 | 13.4 | 74.7 | 697.56 | 28.6 | 20.3 | 66.8 | 701.48 |
|  | 10 | 05 Mar-11 Mar | 29.4 | 11.6 | 63.0 | 8.2 | 865.32 | 28.8 | 12.2 | 69.7 | 681.14 | 30.4 | 12.6 | 70.2 | 685.45 | 28.8 | 20.4 | 65.8 | 692.14 |
|  | 11 | 12 Mar-18 Mar | 31.9 | 12.8 | 57.4 | 7.7 | 875.65 | 30.9 | 12.9 | 62.5 | 673.26 | 32.7 | 13.5 | 64.3 | 688.35 | 28.7 | 20.1 | 63.2 | 693.03 |
|  | 12 | 19 Mar-25 Mar | 31.5 | 14.9 | 54.9 | 8.0 | 898.25 | 30.8 | 14.9 | 60.3 | 682.34 | 32.6 | 16.1 | 61.8 | 692.38 | 29.6 | 20.8 | 67.8 | 701.23 |
|  | 13 | 26 Mar-01 Apr | 34.8 | 15.5 | 48.8 | 9.3 | 845.67 | 33.6 | 15.8 | 53.4 | 632.19 | 34.6 | 16.8 | 54.8 | 645.36 | 30.5 | 21.3 | 64.8 | 643.25 |
|  | 14 | 02 Apr-08 Apr | 35.9 | 18.9 | 47.8 | 7.7 | 912.32 | 34.7 | 19.0 | 52.7 | 693.65 | 35.2 | 19.7 | 53.7 | 688.25 | 30.6 | 22.3 | 63.8 | 672.32 |
|  | 15 | 09 Apr-15 Apr | 33.9 | 17.8 | 50.9 | 6.8 | 875.62 | 33.0 | 18.1 | 56.7 | 602.35 | 32.9 | 18.3 | 58.7 | 576.32 | 30.4 | 22.0 | 65.7 | 568.12 |
|  | 16 | 16 Apr-22 Apr | 38.1 | 21.2 | 37.1 | 6.9 | 813.35 | 36.7 | 22.0 | 43.1 | 587.69 | 37.6 | 22.1 | 45.6 | 536.47 | 31.2 | 22.6 | 66.7 | 532.14 |
|  | 17 | 23 Apr-29 Apr | 38.2 | 21.4 | 41.7 | 8.7 | 895.22 | 36.9 | 21.5 | 46.7 | 597.48 | 37.9 | 22.5 | 47.4 | 574.13 | 31.5 | 21.8 | 64.7 | 547.89 |
|  | 18 | 30 Apr-06 May | 37.1 | 23.0 | 56.0 | 7.8 | 821.05 | 35.9 | 23.6 | 61.2 | 550.14 | 37.2 | 24.6 | 63.3 | 521.66 | 30.2 | 21.3 | 65.2 | 530.20 |
|  | 19 | 07 May-13 May | 38.0 | 22.1 | 45.6 | 7.7 | 741.22 | 36.8 | 22.4 | 51.8 | 502.14 | 37.6 | 23.8 | 50.7 | 407.08 | 31.2 | 22.8 | 66.3 | 401.27 |
|  | 20 | 14 may-20 May | 38.9 | 23.5 | 48.2 | 6.6 | 726.65 | 37.6 | 23.9 | 54.7 | 489.65 | 37.5 | 24.6 | 55.7 | 398.03 | 30.9 | 22.1 | 64.1 | 382.35 |
|  | 21 | 21 May-27 May | 42.6 | 24.0 | 41.7 | 8.9 | 736.22 | 41.3 | 24.7 | 46.7 | 493.37 | 41.3 | 25.7 | 47.9 | 461.29 | 32.5 | 23.5 | 65.3 | 397.81 |
| **Mean** |  |  | **33.32** | **16.49** | **53.41** | **7.4** | **822.28** | **32.3** | **16.84** | **58.74** | **616.00** | **33.73** | **17.71** | **59.98** | **599.81** | **29.71** | **21.11** | **66.03** | **595.55** |
| 2019 | 6 | 05 Feb-11 Feb | 20.9 | 8.5 | 83.9 | 4.3 | 763.65 | 20.2 | 8.9 | 85.6 | 693.26 | 21.6 | 9.2 | 86.3 | 667.53 | 24.8 | 19.3 | 63.2 | 681.46 |
|  | 7 | 12 Feb-18 Feb | 21.4 | 11.3 | 86.5 | 2.5 | 788.25 | 20.8 | 11.6 | 90.2 | 645.67 | 22.1 | 12.0 | 88.6 | 683.65 | 25.6 | 20.1 | 62.4 | 678.65 |
|  | 8 | 19 Feb-25 Feb | 22.9 | 12.0 | 83.0 | 5.0 | 745.63 | 22.1 | 12.5 | 87.6 | 636.47 | 23.3 | 12.6 | 85.8 | 683.17 | 26.9 | 20.8 | 66.4 | 688.86 |
|  | 9 | 26 Feb-04 Mar | 20.9 | 8.9 | 85.1 | 4.6 | 796.32 | 20.2 | 9.3 | 91.3 | 709.45 | 21.4 | 9.7 | 89.4 | 686.28 | 24.7 | 19.6 | 65.7 | 685.47 |
|  | 10 | 05 Mar-11 Mar | 24.4 | 10.0 | 81.1 | 7.7 | 909.55 | 23.6 | 10.3 | 84.5 | 693.27 | 25.4 | 11.2 | 84.5 | 703.65 | 26.7 | 20.4 | 63.2 | 695.45 |
|  | 11 | 12 Mar-18 Mar | 25.4 | 11.4 | 77.5 | 5.9 | 896.62 | 24.5 | 11.9 | 80.3 | 685.35 | 25.9 | 12.1 | 81.4 | 679.56 | 26.3 | 18.4 | 69.7 | 685.26 |
|  | 12 | 19 Mar-25 Mar | 29.9 | 14.1 | 57.8 | 8.7 | 947.66 | 28.6 | 14.8 | 63.5 | 696.47 | 31.2 | 15.3 | 63.7 | 701.49 | 30.2 | 21.6 | 66.5 | 717.35 |
|  | 13 | 26 Mar-01 Apr | 33.0 | 16.4 | 68.3 | 8.6 | 903.27 | 32.1 | 16.6 | 71.8 | 662.78 | 33.8 | 17.6 | 73.2 | 660.45 | 31.8 | 22.8 | 63.1 | 635.78 |
|  | 14 | 02 Apr-08 Apr | 35.8 | 18.2 | 66.4 | 8.8 | 869.63 | 34.9 | 18.9 | 69.5 | 709.17 | 36.4 | 19.1 | 72.1 | 701.28 | 30.8 | 23.5 | 68.4 | 596.46 |
|  | 15 | 09 Apr-15 Apr | 37.4 | 21.5 | 57.9 | 8.3 | 886.63 | 36.5 | 22.1 | 61.7 | 637.78 | 38.6 | 22.3 | 63.5 | 543.98 | 29.1 | 22.4 | 67.8 | 577.38 |
|  | 16 | 16 Apr-22 Apr | 33.9 | 21.6 | 63.4 | 7.0 | 846.38 | 33.2 | 21.8 | 68.9 | 561.45 | 34.8 | 22.8 | 68.7 | 542.64 | 28.6 | 21.4 | 70.1 | 529.24 |
|  | 17 | 23 Apr-29 Apr | 41.3 | 25.4 | 43.7 | 9.3 | 902.47 | 40.3 | 25.9 | 53.1 | 609.78 | 42.2 | 26.3 | 48.9 | 577.33 | 30.1 | 23.5 | 69.2 | 538.15 |
|  | 18 | 30 Apr-06 May | 40.5 | 23.2 | 47.8 | 8.9 | 809.13 | 39.7 | 23.7 | 53.6 | 547.78 | 41.6 | 24.7 | 51.3 | 532.17 | 29.8 | 20.8 | 65.3 | 498.16 |
|  | 19 | 07 May-13 May | 41.4 | 24.6 | 47.9 | 7.1 | 763.45 | 40.3 | 25.1 | 52.8 | 511.45 | 42.3 | 25.9 | 50.2 | 442.36 | 29.5 | 21.3 | 64.1 | 368.15 |
|  | 20 | 14 may-20 May | 36.8 | 22.9 | 57.4 | 6.6 | 702.78 | 36.2 | 23.5 | 56.4 | 481.45 | 38.7 | 23.9 | 61.4 | 432.28 | 28.2 | 20.5 | 63.5 | 367.27 |
|  | 21 | 21 May-27 May | 40.0 | 24.6 | 50.2 | 9.7 | 747.68 | 39.6 | 25.4 | 53.9 | 502.73 | 41.8 | 24.8 | 54.8 | 441.75 | 31.2 | 23.4 | 66.4 | 403.45 |
| **Mean** |  |  | **31.62** | **17.16** | **66.12** | **7.06** | **829.94** | **30.8** | **17.64** | **70.93** | **624.01** | **32.57** | **18.09** | **70.24** | **604.97** | **28.40** | **21.24** | **65.94** | **584.16** |
| 2020 | 32 | 06 Aug-12 Aug | 35.8 | 27.7 | 76.7 | 5.1 | 923.14 | 35.1 | 27.9 | 80.1 | 772.45 | 36.5 | 26.7 | 82.3 | 666.45 | 25.4 | 24.6 | 67.8 | 678.29 |
|  | 33 | 13 Aug-19 Aug | 33.6 | 26.6 | 83.5 | 3.7 | 906.35 | 33.2 | 26.8 | 85.6 | 763.28 | 33.2 | 26.1 | 84.6 | 657.45 | 26.2 | 25.8 | 66.5 | 666.35 |
|  | 34 | 20 Aug-26 Aug | 31.9 | 25.9 | 83.3 | 4.1 | 896.35 | 30.4 | 26.1 | 86.2 | 725.84 | 32.4 | 25.1 | 86.4 | 636.84 | 26.8 | 25.4 | 69.2 | 672.45 |
|  | 35 | 27 Aug-02 Sept | 32.9 | 26.3 | 77.4 | 4.5 | 876.25 | 32.2 | 26.8 | 81.4 | 698.45 | 32.4 | 25.8 | 80.3 | 609.65 | 27.3 | 26.3 | 66.3 | 623.78 |
|  | 36 | 03 Sept-09 Sept | 34.3 | 26.0 | 78.4 | 6.4 | 896.25 | 33.6 | 26.4 | 84.3 | 730.65 | 33.8 | 25.3 | 83.7 | 632.14 | 27.9 | 25.2 | 64.8 | 636.31 |
|  | 37 | 10 Sept-16 Sept | 35.6 | 25.8 | 72.6 | 5.5 | 902.35 | 34.8 | 26.1 | 78.4 | 746.38 | 35.2 | 25.3 | 80.2 | 642.57 | 28.2 | 23.4 | 66.3 | 623.65 |
|  | 38 | 17 Sept-23 Sept | 37.1 | 24.8 | 68.6 | 7.8 | 878.18 | 36.5 | 25.3 | 73.2 | 713.46 | 36.1 | 23.4 | 76.4 | 602.58 | 26.6 | 23.5 | 64.6 | 598.47 |
|  | 39 | 24 Sept-30 Sept | 35.4 | 23.1 | 68.5 | 7.1 | 745.38 | 34.9 | 23.7 | 72.4 | 691.28 | 34.8 | 22.3 | 75.4 | 596.35 | 26.5 | 22.8 | 65.9 | 591.45 |
|  | 40 | 01 Oct-07 Oct | 35.4 | 20.3 | 61.5 | 8.9 | 701.24 | 34.5 | 20.8 | 66.3 | 671.34 | 34.9 | 21.4 | 68.9 | 568.95 | 26.8 | 22.7 | 67.3 | 545.36 |
|  | 41 | 01 Oct-07 Oct | 35.0 | 19.3 | 61.2 | 8.1 | 679.32 | 34.3 | 20.6 | 63.7 | 646.38 | 34.6 | 21.4 | 67.5 | 545.32 | 25.1 | 23.4 | 64.4 | 532.12 |
|  | 42 | 08 Oct-14 Oct | 34.2 | 17.7 | 58.3 | 6.0 | 699.28 | 33.6 | 18.6 | 64.2 | 632.73 | 35.4 | 19.6 | 68.3 | 521.36 | 25.3 | 22.7 | 67.3 | 501.47 |
|  | 43 | 15 Oct-21 Oct | 33.2 | 13.2 | 58.1 | 5.7 | 704.22 | 32.1 | 13.3 | 63.8 | 602.56 | 34.2 | 18.8 | 67.2 | 501.32 | 26.3 | 22.3 | 65.7 | 487.43 |
|  | 44 | 22 Oct-28 Oct | 30.2 | 10.8 | 57.9 | 5.0 | 687.32 | 29.7 | 11.2 | 62.7 | 596.45 | 31.2 | 20.3 | 65.2 | 486.65 | 25.4 | 22.7 | 66.2 | 471.45 |
|  | 45 | 29 Oct-04 Nov | 29.1 | 10.6 | 58.5 | 2.4 | 765 | 28.5 | 11.0 | 62.9 | 643.45 | 29.1 | 18.6 | 65.4 | 579.68 | 26.7 | 21.8 | 67.8 | 475.65 |
|  | 46 | 05 Nov-11 Nov | 27.5 | 11.3 | 64.1 | 1.6 | 786.39 | 26.8 | 11.8 | 70.1 | 632.24 | 26.4 | 17.6 | 71.2 | 563.24 | 27.6 | 20.6 | 66.2 | 435.45 |
|  | 47 | 12 Nov-18 Nov | 24.7 | 7.9 | 57.9 | 5.6 | 745.33 | 24.0 | 8.6 | 66.4 | 598.65 | 25.4 | 16.8 | 67.3 | 521.32 | 25.8 | 20.8 | 67.4 | 401.45 |
|  | 48 | 19 Nov-25 Nov | 24.8 | 10.1 | 58.6 | 5.9 | 736.25 | 23.9 | 10.8 | 66.8 | 575.36 | 25.3 | 17.9 | 67.5 | 511.30 | 26.3 | 19.6 | 67.7 | 365.45 |
| **Mean** |  |  | **32.39** | **19.26** | **67.36** | **5.49** | **795.80** | **31.65** | **19.75** | **72.26** | **627.99** | **32.41** | **21.91** | **73.99** | **579.01** | **26.48** | **23.15** | **66.55** | **547.54** |

SMW = Standard Meteorological Week; OF = Open field; IPNH = Insect proof net house; NVPH = Naturally ventilated polyhouse; HTGH = High-tech green house; RH = Relative Humidity (%); BSS (hr) = Bright sunshine (hours); Light Intensity (LI) was calculated as Photosynthetic Active Radiation (PAR)

**Table 2.** Parameter estimates of regression models of yield on different traits

| **Regression of yield on yield contributing traits** | | | | | | |
| --- | --- | --- | --- | --- | --- | --- |
| Term | Estimate | Std Error | t Ratio | Prob>\|t\| | Lower 95% | Upper 95% |
| Intercept | -128.8852 | 134.5877 | -0.96 | 0.3405 | -395.8392 | 138.06884 |
| FL | 37.518698 | 8.702481 | 4.31 | <.0001* | 20.257369 | 54.780027 |
| FD | -80.43658 | 25.37691 | -3.17 | 0.0020* | -130.7716 | -30.1016 |
| FW | 7.7403514 | 0.667126 | 11.60 | <.0001* | 6.4171093 | 9.0635934 |
| F/P | 22.884872 | 15.06485 | 2.52 | 0.0418** | -6.996195 | 52.765938 |
| **Regression of yield on earliness** | | | | | | |
| Term | Estimate | Std Error | t Ratio | Prob>\|t\| | Lower 95% | Upper 95% |
| Intercept | 4278.1491 | 146.8352 | 29.14 | <.0001* | 3986.9693 | 4569.3288 |
| NNFFF | -48.47962 | 8.679988 | -5.59 | <.0001* | -65.69236 | -31.26687 |
| DFFFA | -12.51547 | 5.783482 | -2.16 | 0.0328* | -23.98433 | -1.046604 |
| DFFH | -33.35154 | 4.656351 | -7.16 | <.0001* | -42.58526 | -24.11782 |
| **Regression of yield on mineral traits** | | | | | | |
| Term | Estimate | Std Error | t Ratio | Prob>\|t\| | Lower 95% | Upper 95% |
| Intercept | -29.908 | 141.6555 | -0.21 | 0.8332 | -310.881 | 251.06498 |
| P | 2.9351187 | 3.557767 | 0.82 | 0.4113 | -4.121695 | 9.9919319 |
| K | -0.899878 | 0.514208 | -1.75 | 0.0831 | -1.919807 | 0.1200499 |
| Mn | 25.07775 | 7.072797 | 3.55 | 0.0006* | 11.048891 | 39.10661 |
| Zn | 60.851743 | 15.09366 | 4.03 | 0.0001* | 30.913539 | 90.789946 |
| Fe | 50.456174 | 9.750502 | 5.17 | <.0001* | 31.116101 | 69.796248 |
| **Regression of yield on antioxidants** | | | | | | |
| Term | Estimate | Std Error | t Ratio | Prob>\|t\| | Lower 95% | Upper 95% |
| Intercept | -185.9129 | 133.5546 | -1.39 | 0.1670 | -450.8814 | 79.055589 |
| JC | 8.1219764 | 3.206218 | 2.53 | 0.0129* | 1.7609314 | 14.483021 |
| DPPH | 5.2733684 | 3.961844 | 1.33 | 0.1862 | -2.586817 | 13.133554 |
| Chl | 16.265797 | 27.63248 | 0.59 | 0.5574 | -38.55627 | 71.087859 |
| Vit_C | 6.5172941 | 3.774468 | 1.73 | 0.0873 | -0.971143 | 14.005731 |
| Carot | 15.082179 | 4.8968 | 3.08 | 0.0027* | 5.3670669 | 24.797291 |
| Sap | -0.43764 | 0.580016 | -0.75 | 0.4523 | -1.588376 | 0.7130963 |
| Charan | 0.760409 | 5.079788 | 0.15 | 0.8813 | -9.317746 | 10.838564 |
| * indicates significant at 1 % level of significance  ** indicates significant at 1 % level of significance | | | | | | |

**Table 3.** PCA on earliness

| **Eigenvalues** | | | **Eigenvectors** | | | |
| --- | --- | --- | --- | --- | --- | --- |
| **No.** | **Eigenvalue** | **Percent** | **Trait** | **PC1** | **PC2** | **PC3** |
| 1 | 2.6462 | 88.208 | NNFFF | 0.58531 | -0.27353 | -0.76328 |
| 2 | 0.2219 | 7.397 | DFFFA | 0.57938 | -0.51746 | 0.62973 |
| 3 | 0.1319 | 4.396 | DFFH | 0.56721 | 0.81081 | 0.14440 |

**Table 4.** PCA on minerals

| **Eigenvalues** | | | **Eigenvectors** | | | | | |
| --- | --- | --- | --- | --- | --- | --- | --- | --- |
| **No.** | **Eigenvalue** | **Percent** | **Trait** | **PC1** | **PC2** | **PC3** | **PC4** | **PC5** |
| 1 | 2.3937 | 47.875 | P | 0.52780 | -0.46962 | -0.10143 | -0.00619 | -0.70040 |
| 2 | 1.2059 | 24.118 | K | 0.49072 | -0.49610 | 0.26158 | -0.04953 | 0.66498 |
| 3 | 1.1116 | 22.232 | Mn | 0.31835 | 0.42269 | 0.66742 | 0.50360 | -0.14462 |
| 4 | 0.1750 | 3.501 | Zn | 0.48439 | 0.54102 | -0.05316 | -0.68526 | 0.01603 |
| 5 | 0.1137 | 2.274 | Fe | 0.38031 | 0.24895 | -0.68775 | 0.52375 | 0.21463 |

**Table 5.** PCA on antioxidants

| **Eigenvalues** | | | **Eigenvectors** | | | | | | | |
| --- | --- | --- | --- | --- | --- | --- | --- | --- | --- | --- |
| **No.** | **Eigenvalue** | **Percent** | **Trait** | **PC1** | **PC2** | **PC3** | **PC4** | **PC5** | **PC6** | **PC7** |
| 1 | 4.281 | 61.154 | JC | 0.457 | 0.105 | -0.008 | 0.541 | -0.275 | 0.542 | -0.343 |
| 2 | 1.259 | 17.985 | DPPH | 0.373 | 0.459 | -0.301 | -0.142 | -0.447 | -0.134 | 0.567 |
| 3 | 1.029 | 14.699 | Chl | 0.329 | -0.310 | 0.593 | -0.353 | -0.491 | -0.218 | -0.178 |
| 4 | 0.174 | 2.489 | Vit_C | 0.434 | 0.264 | -0.084 | -0.620 | 0.456 | 0.256 | -0.279 |
| 5 | 0.112 | 1.594 | Carot | 0.461 | 0.012 | -0.092 | 0.363 | 0.316 | -0.715 | -0.191 |
| 6 | 0.088 | 1.258 | Sap | -0.073 | 0.581 | 0.723 | 0.191 | 0.239 | 0.019 | 0.202 |
| 7 | 0.057 | 0.820 | Charan | 0.372 | -0.525 | 0.141 | 0.100 | 0.345 | 0.253 | 0.611 |

**Table 6.** PCA on yield contributing traits

| **Eigenvalues** | | | **Eigenvectors** | | | | |
| --- | --- | --- | --- | --- | --- | --- | --- |
| **No.** | **Eigenvalue** | **Percent** | **Trait** | **PC1** | **PC2** | **PC3** | **PC4** |
| 1 | 2.3321 | 58.301 | FL | 0.46110 | 0.67815 | 0.33765 | 0.46206 |
| 2 | 0.8430 | 21.076 | FD | 0.51030 | 0.28979 | -0.68233 | -0.43594 |
| 3 | 0.5994 | 14.986 | FW | 0.49968 | -0.60466 | -0.24669 | 0.56907 |
| 4 | 0.2255 | 5.637 | F/P | 0.52660 | -0.30086 | 0.59964 | -0.52212 |

**Table 7.** Parameter estimates of regressing yield on first principal components of earliness, minerals, antioxidants and yield contributing traits

| **Term** | **Estimate** | **Std Error** | **t Ratio** | **Prob>\|t\|** |
| --- | --- | --- | --- | --- |
| Intercept | 1649.6862 | 12.86915 | 128.19 | <.0001* |
| Earliness PC1 | -164.5985 | 24.04565 | -6.85 | <.0001* |
| Antioxidants PC1 | 37.154909 | 21.11757 | 1.76 | 0.0815 |
| Minerals PC1 | -23.31227 | 15.55997 | -1.50 | 0.1372 |
| yield contributing traits PC1 | 36.865059 | 17.70752 | 2.08 | 0.0399* |
| * indicates significant at 1 % level of significance | | | | |
